# Plasmon Enhanced Universal SERS Detection of Hierarchical Plastics by 3D Plasmonic Funnel Metastructure

**DOI:** 10.1002/advs.202500062

**Published:** 2025-05-08

**Authors:** Weixi Lu, Jian Luo, Yuyang Zhuang, Jie Liang, Min Xiong, Hui Liu, Lin Zhou

**Affiliations:** ^1^ College of Engineering and Applied Sciences Nanjing University Nanjing 210023 China; ^2^ School of Physics and Optoelectronic Engineering Yangtze University Jingzhou 434023 China

**Keywords:** concentration, hierarchical, micro‐nano plastics, plasmonic hot spots, SERS

## Abstract

Plasmonic nanostructures have aroused tremendous excitement in extreme light matter interactions because of efficient light harvesting and nanometer field concentration, ideal for solar thermal conversion, photocatalysis, photodetection, etc. Here a 3D self‐assembled plasmonic nanostructure is reported for ultrasensitive SERS detection of hierarchical micro‐nano plastic pollutants ranging from 30 nm to microns by rationally integrating high density of both surface and volumetric hot spots into one structure, enabled by V‐shaped close‐packed bi‐metallic nanoparticles with massive nanovoids across transverse and longitudinal areas. The unique bi‐metallic structure of hollow nanocones can enable an enhancement factor up to 1.1 × 10^8^ as well as self‐built enrichment of targeting hierarchical analytes toward the size‐matched hot spot areas, resulting in not only race detection of micro‐nano plastics with concentration down to 10^−8^ g L^−1^ but also universal adaptability to simultaneous detection of a broad range of pollutants beyond micro‐nano plastics. The results offer a practical solution for trace detection of hierarchical micro‐nano plastics and other mixed aqueous pollutants, demonstrating considerable potential for combating water pollution.

## Graphical abstract

A 3D self‐assembled plasmonic nanostructure for ultrasensitive SERS detection of hierarchical micro‐nano plastic pollutants ranging from 30 nm to microns by rationally integrating high density of both surface and volumetric hot spots into one structure, enabled by V‐shaped close‐packed bi‐metallic nanoparticles with massive nanovoids across transverse and longitudinal areas. The unique bi‐metallic structure of hollow nanocones can enrich the targeting hierarchical analytes toward the size‐matched hot spot areas, resulting in not only race detection of micro‐nano plastics with concentration down to 10^8^ g L^−1^, but also universal adaptability to simultaneous detection of a broad range of pollutants beyond micro‐nano plastics.

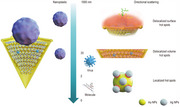

## 1 Introduction

Plastic exposure has become one of the most urgent safety risks for humanity nowadays,^[^ ^1^, ^2^, ^3^, ^4^, ^5^, ^6^, ^7^, ^8^ ^]^ where micro‐nano plastics with hierarchical geometry sizes (<5 µm) and large specific volume‐to‐surface‐area ratio have been receiving increasing attention^[^ ^5^, ^6^ ^]^ because of the ideal carrier nature for harmful analytes such as virus and/or bacteria. However, as most plastic residuals in versatile aqueous conditions are featured by versatile particle sizes as well as the relatively inert chemical/physical properties, efficient detection of micro‐nano plastic pollutants is urgently needed but rather challenging.

The combination of plasmonic nanostructures with surface‐enhanced Raman scattering (SERS) has evidenced the powerful capability of ultrasensitive photodetection in the past decades.^[^ ^7^, ^8^, ^9^, ^10^, ^11^, ^12^, ^13^, ^14^, ^15^, ^16^ ^]^ Massive plasmonic metasurfaces with planar hot spot effects have been developed and widely employed as plasmonic SERS substrates,^[^ ^12^, ^15^, ^16^, ^17^, ^18^, ^19^, ^20^, ^21^, ^22^ ^]^ which are quite convenient for operation primarily for targeting analytes with relatively homogeneous constituents and/or geometry structures. Very recently, 3D plasmonic nanostructures, ^[^ ^23^, ^24^, ^26^, ^27^, ^28^, ^29^, ^30^ known as the flexible capability of spectral manipulation as well as high density of stereo‐aligned hot spots or field localization, have shown particular relevance to efficient light harvesting for solar steam generation,^[^ ^25^, ^27^ ^]^ water purification and plasmonic monitoring.^[^ ^31^, ^32^ ^]^ 3D nanostructures have shown great promise in SERS based enhanced photodetection in a variety of small molecules such as dyes and drugs.^[^ ^12^, ^21^ ^]^ However, efficient plasmonic SERS detection of hierarchically targeted analytes, such as aqueous micro‐nano plastics, encounters severe difficulties. The scale of plastics is limited to no more than 300 nm. The inefficient detection of micro‐scale plastics by the reported 3D plasmonic SERS substrates is induced by the small hotspot region less than 5 nm.^[^ ^9^, ^10^, ^33^, ^34^ ^]^ To address the significant spatial mismatch between targeted plastic pollutants and hotspot regions, a novel optical design is necessary.

In this work, we have demonstrated a hierarchical 3D plasmonic nanostructure composed of bi‐metallic hollow nanocones (BHNC). Three types of near‐field hot spots are rationally designed to coexist in the structure, with typical sizes well covering the range from nanometer to micrometer, enabling pronounced plasmon enhanced SERS signals over versatile micro‐nano plastic pollutants. Besides the high performance in the generation of SERS signals, the far‐field scattering profiles are well regulated by this structure and thus the collection efficiency of SERS signals can be significantly increased, further improving the detection limit. This work provides practical routes to the ultrasensitive detection of complicated micro‐nano pollutants with heterogeneous sizes and/or constituents.

**Figure 1:**
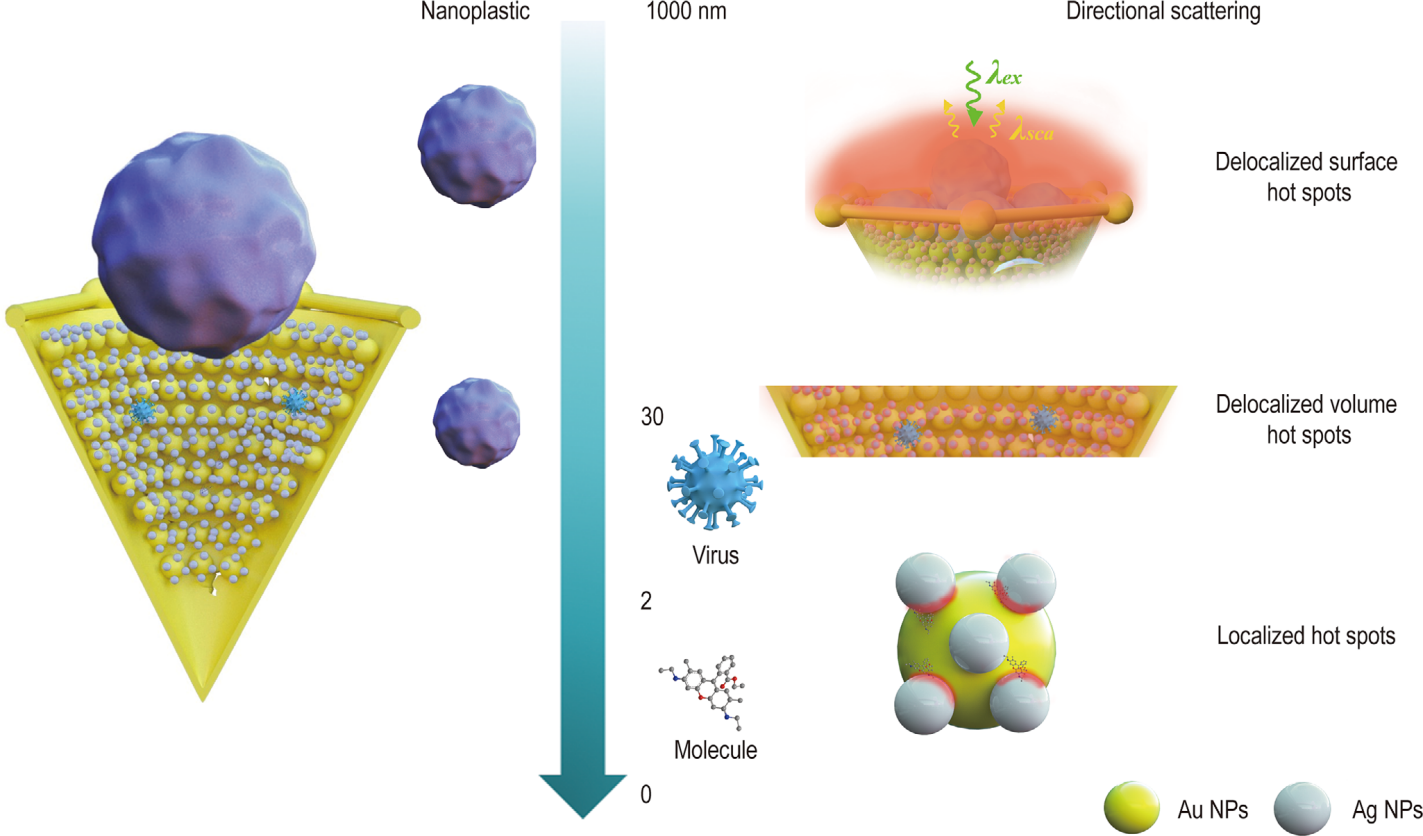
Schematic of the BHNC structure and detection for micro‐nano plastics and other pollutants from sub‐nanometer‐to‐micrometer scales.

The proposed BHNC structure for plasmonic SERS substrate is schematically illustrated in **Figure** **1**. To enable the efficient SERS detection of hierarchical micro‐nano pollutants with poor optical detectability and/or low aqueous concentration, there are at least three physical effects at work in this structure. First, hierarchical hot spot areas ranging from nanometer to micrometer scale are needed to effectively capture various‐sized pollutants in contaminated water. Second, the targeting hierarchical pollutants should be efficiently concentrated into the size‐matched near‐field hot spot areas to maximize SERS signals. Third, in order to improve the detection efficiency, the structure should be favorable for directionally backward light scattering in a wide spectral range.

The proposed BHNC structure in Figure 1 can serve as the ideal candidate to fully address the above requirements with two crucial structural features: 1) The close‐packed bi‐metallic plasmonic nanoparticles self‐assembled in vertical directions can provide 3D high‐density hot spots distributed in different host areas. As depicted in the right panel of Figure 1, the BHNC involves the delocalized surface hot spots (DSHSs) near the top of the SERS structure, the delocalized volumetric hot spots (DVHSs) generated by the cavity resonances inside the void zones of nanocones, as well as the localized hot spots (LHSs) close to the dense nanogaps among metallic nanoparticles. 2) The nanoparticles are formed in a well‐defined graded V‐type profile to enhance light matter interactions and regulate the broadband back scattering ideal for vast pollutant detection. In addition, pollutants of different sizes can be distinguished and accumulated in hot spot areas with similar sizes. Therefore, the proposed BHNC structure is capable of ultrasensitive detection ranging from small drug molecules (sub‐nanometer), and viruses (tens nanometers) to micro‐nano plastic pollutants.

**Figure 2:**
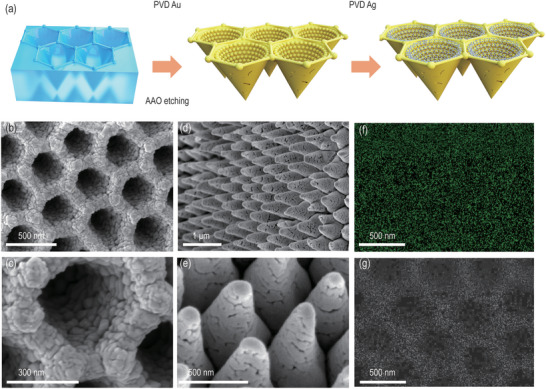
Characterization of the fabrication of the bi‐metallic BHNC (noted as BHNC‐Au/Ag) structure. a) Fabrication process b,c) top‐view and d,e) 3D cross‐sectional SEM images of the prepared BHNC‐Au/Ag, respectively. f,g) EDS elemental maps of Au (f) and Ag (g) on the BHNC‐Au/Ag.

To reproduce the proposed 3D plasmonic nanostructure in the experiment, a sequential physical vapor deposition (PVD) procedure of gold (Au) and silver (Ag) nanoparticles (noted as BHNC ‐Au/Ag) on a V‐typed nanoporous‐template (NPT) is employed for the fabrication (shown in **Figure** **2**a), followed by an NPT removal procedure for the desired nanopore exposure. The sacrificed V‐shaped NPT is employed for the self‐assembly of the 3D porous nanoparticles full of hierarchical hot spot zones while the heterogeneous bi‐metallic (instead of single metal) nanoparticles are introduced to further boost the local field enhancement. The deposited thickness of Au and Ag were optimized in order to generate substance accumulation channels (Figure S1, Supporting Information) as well as a high density of nanogaps for localized hotspots (LHSs) (Figure S2, Supporting Information), as demonstrated by a representative sample with 250 nm‐thick Au and 30 nm‐thick Ag (noted as BHNC‐Au/Ag). The microscopic surface and volumetric morphology of the structure were then characterized by scanning electron microscopy (SEM) and energy‐dispersive X‐ray spectroscopy (EDS), as shown in Figure 2b–g. The 3D self‐assembled bimetallic nanoparticles are close‐packed in the hollow nanocones profile favorable for high density of nanogaps (see more details in Figure S3 (Supporting Information) for the statistical distributions of nanoparticle sizes), enabling the generation of high‐density hot spots at hierarchical geometry sizes. The SEM images of the BHNC‐Au/Ag suggest the formation of the self‐assembled V‐type nanocones composed of nanoparticles with nanometer‐sized intergaps on the sidewalls (Figure 2b–e). The EDS elemental maps further confirm the homogenous distribution of Au and Ag metals in this structure (Figure 2f,g). The 3D porous nanocone structure can effectively enhance light‐matter interactions, modulate the operation bandwidth of surface plasmons, and simultaneously regulate the backscattering responses. By carefully manipulating the PVD condition, the intergap of nanocone can be well controlled. Here the average intergap size of the prepared 3D BHNC‐Au/Ag structure is ≈20 nm (retrieved from the statistical distribution in Figure S4, Supporting Information), beneficial for the target molecule enrichment at targeted locations for enhanced SERS detection of ultralow concentrated pollutants. The detailed cross‐sectional SEM image (Figure S5, Supporting Information) further reveals the fine structure of a connected Au film underneath the high density of Au NPs, indicating the mechanical stability of the free‐standing porous structure.

**Figure 3:**
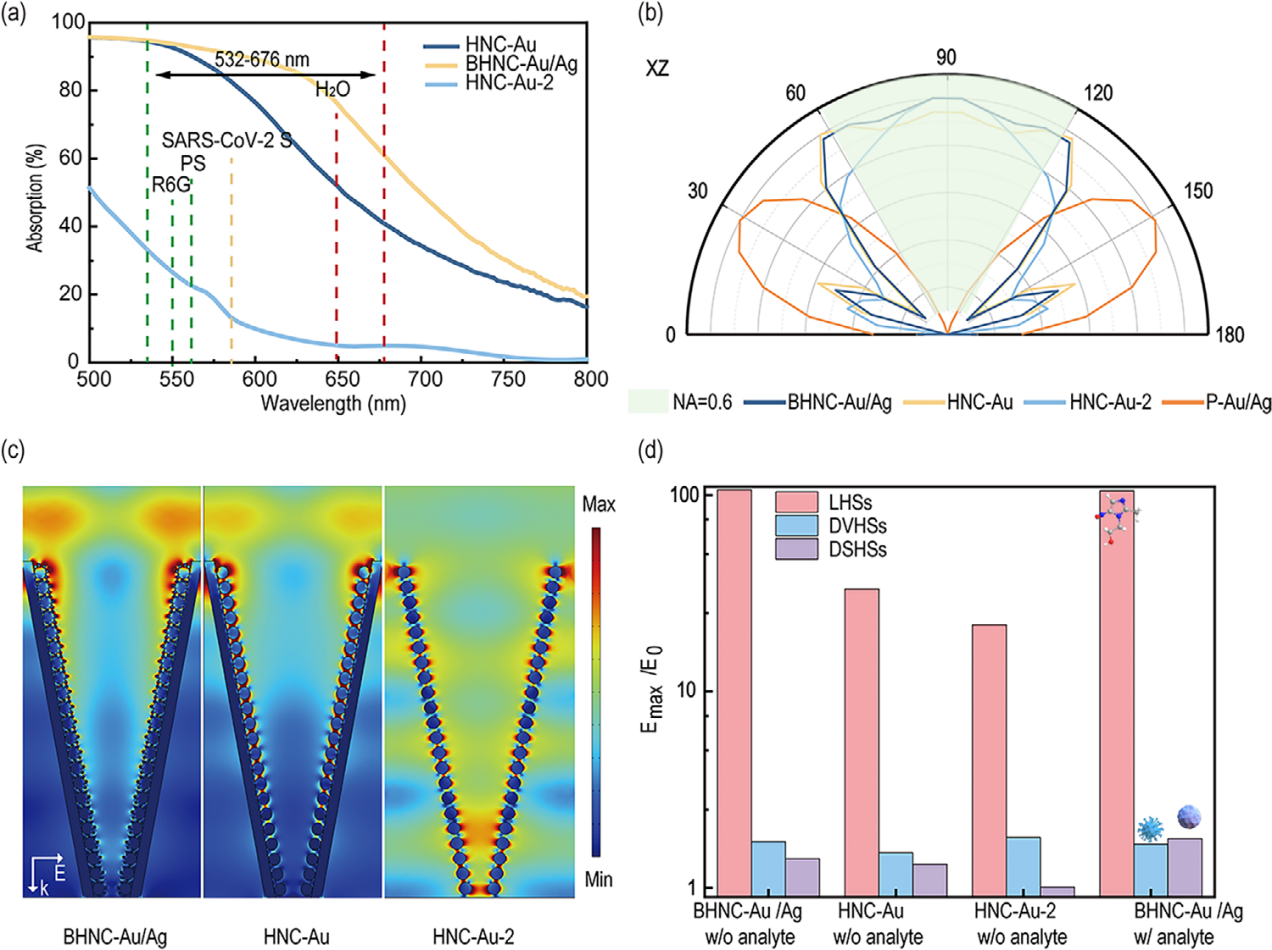
Far‐field and near‐field optical responses of different BHNC structures. a) The far‐field absorption spectra of BHNC‐Au/Ag, HNC‐Au (250 nm) (noted as HNC‐Au), HNC‐Au (80 nm) (noted as HNC‐Au‐2), and P‐Au/Ag structures were characterized and the characteristic Raman scattering wavelengths of different analytes are listed. b) The far‐field radiation patterns of BHNC‐Au/Ag, HNC‐Au, HNC‐Au‐2, and P‐Au/Ag were calculated, with all patterns normalized for comparison. c) Near‐field electric field distribution views for BHNC‐Au/Ag, HNC‐Au, and HNC‐Au‐2 at a wavelength of 532 nm. d) Maximum hot spot intensities were observed for the HNC structures, corresponding to the detection of small drug molecules (MNZ), SARS‐CoV‐2 S proteins, and nanoplastics in the BHNC‐Au/Ag structure, respectively.

To evaluate the SERS performance of the BHNC structures, its far‐field and near‐field optical properties were characterized, as illustrated in **Figure** **3**. Figure 3a experimentally demonstrates the light harvesting performance of the BHNC structure, and the optimization of the nanocone's structural parameters is partially described in Figures S6 and S7 (Supporting Information). As shown, the BHNC‐Au/Ag structure demonstrates broad‐spectrum plasmonic light harvesting capabilities in the range from 500 to 700 nm, indicating that the structure can enhance light‐matter interactions across a broad range of Raman excitation and scattering wavelengths. Which is profiting from the 3D self‐assembled funnel structure. These results indicate the excellent far‐field light harvesting capability of the BHNC structure on the generation and collection of Raman signals.

The far‐field direction‐dependent backscattering patterns calculated by the 3D finite difference‐time domain (FDTD) method confirm the advantage of the proposed BHNC structures on the collection efficiency of scattering light over single metallic hollow nanocones (HNC) and planar‐Au/Ag(P‐Au/Ag) plasmonic structures. Figure 3b shows the backscattering patterns of HNC structures and P‐Au/Ag. In these calculations, one nanoplastic particle was placed at the center areas of these structures. As shown in Figure 3b, while the Raman scattering angle of the P‐Au/Ag structure is within the range of ±82°, significantly exceeding the collection angle of commonly used Raman instruments, the HNC structure maintains consistency with the collection angle, with the BHNC‐Au/Ag structure exhibiting the best performance, featuring a Raman backscattering angle of ±39° and a collection efficiency 1.2 times higher than that of the Au/Ag structure. The above demonstrates the ability of the BHNC structure to achieve Raman scattering light collimation.

Furthermore, the finite element method (FEM) calculations demonstrated the advantage of SERS enhancement of the BHNC structures in the Near‐field. Figure 3b shows the calculated electric field distributions of these HNC structures under 532 nm excitation. The electric field distribution for the BHNC‐Au/Ag exhibits effective light confinement within the hollow cavity. The maximum local field strength enhancement factor E_max_/E_0_ was 106.3. Notably, three distinct hot spots are identified: DSHSs located at the top, DVHSs within the cavity, and LHSs at the surface of the NPs, respectively. The generation of DSHSs and DVHSs can be attributed to the optical field coupling within the cavity, facilitated by backscattering from the connected Au film interacting with the Au NPs and Ag NPs. Conversely, LHSs generated by the curved surfaces of NPs on the walls and bottom of the structure mainly stemmed from near‐field coupling between adjacent Au and Ag NPs, as well as the connected Au film. Hierarchical pollutants with different sizes ranging from nanometers to micrometers deposit on different hot spots, and thus all of their SERS signals can be enhanced. Comparatively, the light confinement of the HNC‐Au‐2 structure is rather poor. Severe light leakage occurs and thus a large number of DVHSs areas are generated, which is inaccessible for most hierarchical plastic particles. The light confinement can be significantly enhanced by increasing the deposited Au thickness to 250 nm in both the inner cavity zone and the surface, but still weaker than in HNC‐Au/Ag. Quantitatively, although the enhancement factors of DSHS and DVHS between in HNC‐Au and HNC‐Au/Ag are similar, the enhancement factor of LHS in the latter is ≈3.2 folds larger than the latter (Figure 3c). This confirms the optimization of bi‐metallic in LHSs enhancement and thus the Raman performance (Figure S8, Supporting Information). To evaluate the impact of target pollutants on light field distribution upon entering the cavity, we analyzed the effects of nanoplastics (500 nm), SARS‐CoV‐2 S proteins (20‐30 nm),^[^ ^35^ ^]^ and drug molecules^[^ ^36^, ^37^ ^]^ (MNZ, sub‐nanometer) on the structural light field (Figure S9, Supporting Information), respectively. Figure 3d illustrates the little impact of drug molecules and SARS‐CoV‐2 S proteins on the near‐field enhancement of BHNC‐Au‐Ag. In contrast, Micro‐nano plastics cause an increased DSHSs density since their typical sizes are comparable to the incident light wavelength and then cause a redistribution of the light field. A similar effect on the simulated electric field distribution is also found for polystyrene (PS) micro‐nano plastics (Figure S10, Supporting Information). Figure S11 (Supporting Information) further illustrates the universality of the BHNC‐Au‐Ag for various types of micro‐nano plastics and other pollutants in complex environments.

**Figure 4:**
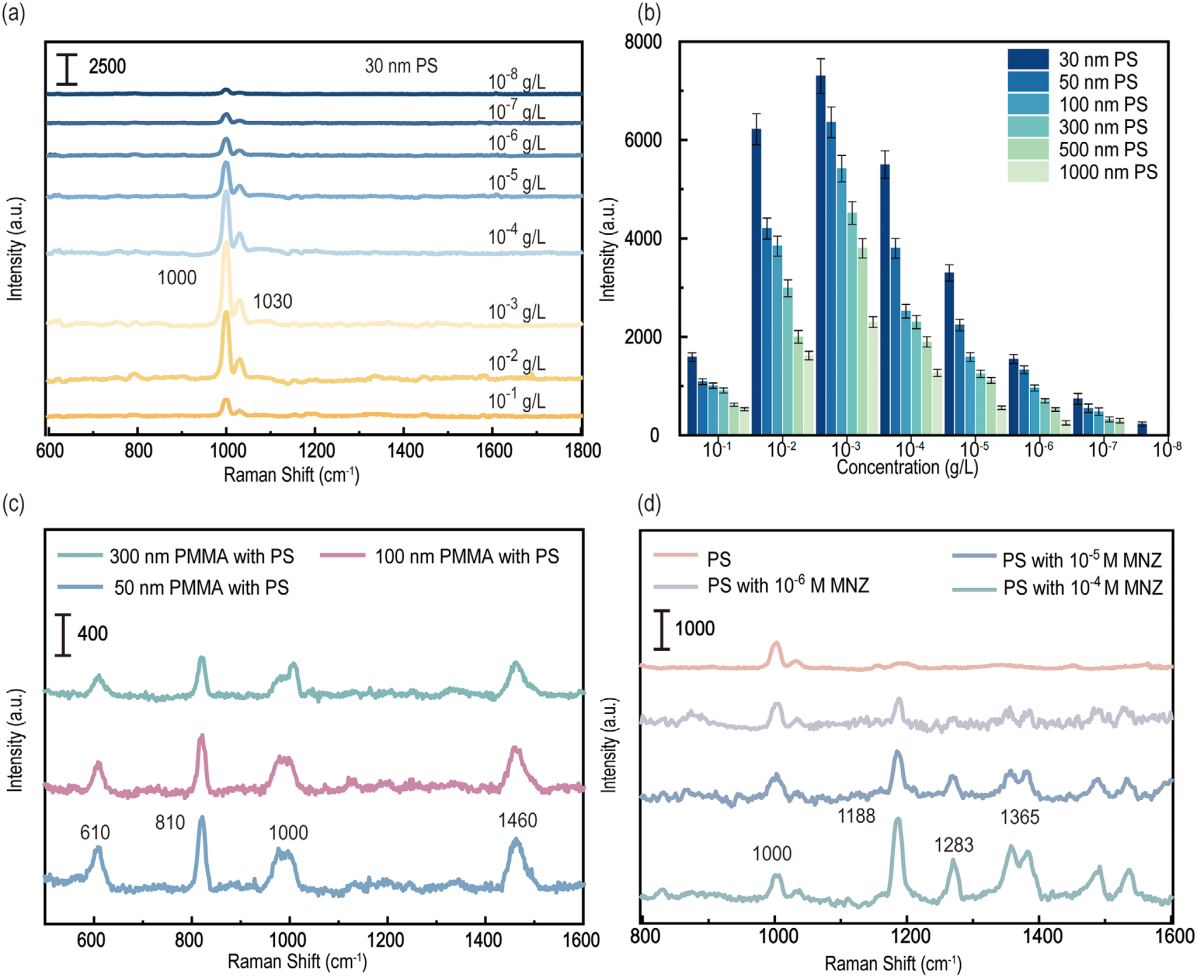
SERS performance of the BHNC structure for analytes detection from sub‐nano to micrometer scales. a) 30 nm PS nanoplastics on BHNC from 10^−1^ to 10^−8^ g L^−1^. The volume of PS solution enrichment was 2 ml. b) 1000 cm^−1^ intensity of 30, 50, 100, 300, 500, and 1000 nm PS micro‐nano plastics solutions with different concentrations. Raman spectra of solutions with different concentration ratios of c) PS (1 µm, 10^−6^ g L^−1^) with PMMA (10^−3^ g L^−1^) from 50 to 300 nm. d) PS (30 nm, 10^−6^ g L^−1^) mixed with MNZ from 10^−4^ to 10^−6^ m.

To validate the crucial roles of hierarchical hot spots and broadband optical responses of the proposed BHNC structures in the detection of multi‐scale hierarchical pollutants, the standard SERS detection procedures were performed for versatile types of analytes, including hierarchical micro‐nano plastics (30 nm to 1 µm), SARS‐CoV‐2 S proteins, small drug molecules, dyes molecules, and mixed solutions of hierarchical plastics with other small molecules, as shown in **Figure** **4**. Additionally, Figure S12 (Supporting Information) shows that the filterability of the BHNC‐Au/Ag structure was 80.1% for 30 nm nanoplastics and over 99% for micro‐nano plastics ranging from 100 to 1000 nm, indicating excellent enrichment capabilities for micro‐nano plastics prior to SERS detection. Figure 4a depicts the Raman spectra of 30‐nm‐sized PS nanoplastics down to 10^−8^ g L^−1^, where 1000 cm^−1^ and 1030 cm^−1^ peaks correspond to the C‐C ring breathing mode and C‐H in‐plane deformation,^[^ ^38^ ^]^ respectively. As the 30‐nm‐sized PS nanoplastics concentration(amounts) surpasses the optimal level, scattering and absorption by the plastics on the surface reduce the laser power in the hot spot region, causing the Raman signal to decrease.^[^ ^8^, ^39^ ^]^ Both PS micro‐nano plastics exhibit a decrease in Raman signal at concentrations exceeding the optimal level. In addition, the BHNC structure was characterized for the detection of multi‐scale pollutants, using micro‐nano plastics (30–1000 nm PS) as an example, as shown in Figure 4b. The SERS signal is detectable for hierarchical micro‐nano plastics ranging from 30 nm to microns even in concentration 10^−8^ g L^−1^, indicating the capability of trace detection. Detailed SEM analysis further confirms the ability of the proposed BHNC‐Au/Ag structure to efficiently capture hierarchically sized micro‐nano plastics in the hot spot regions (Figure S13, Supporting Information). Micro‐nano plastics larger than 450 nm are enriched on the surface of the structure, where their Raman signals are enhanced by DSHSs. Plastics with sizes smaller than 450 nm are preferentially concentrated in the cavity driven by water pressure, thus the SERS signals are enhanced by DVHSs and LHSs. In addition, several common plastics have been examined in Figure S14 (Supporting Information) to further illustrate the generalizability of the structure for plastic detection.

The unique V‐shaped nanoporous nanocone of the HNC structure generates multi‐scale hotspots that are universal for multi‐scale pollutant detection, effectively enhancing Raman signals from analytes. As shown in Figure 4c, the BHNC structure was characterized for the detection of multi‐scale pollutants, using micro‐nano plastics mixture 10^−6^ g L^−1^ PS (1 µm) with 10^−3^ g L^−1^ PMMA (from 50 to 300 nm) as an example. Raman characteristic peaks of PS (1000 cm⁻¹) and PMMA (600, 810, 1460 cm⁻¹) were detected in the mixed solution of PMMA (from 50 to 300 nm) and PS microplastics of various sizes, indicating that the multi‐scale hotspots property of the structure play crucial roles in enhanced Raman signals of highly hierarchical pollutants with different sizes and/or constituent types of micro‐nano plastics.

Unlike previous chemically synthesized enrichment structures,^[^ ^6^, ^8^, ^39^, ^40^ ^]^ the BHNC structures enable simultaneous trace detection of micro‐nano plastics and other contaminants. Figure 4d illustrates the Raman spectra of solutions featuring different ratios between PS (30 nm, 10^−6^ g L^−1^) and MNZ. As the concentration of MNZ increases, its characteristic peaks become more pronounced, while the characteristic peak of PS (1000 cm^−1^) remains constant. Previous studies have demonstrated that chemometric analysis enables the effective identification of multiple analytes, even when their Raman peaks overlap,^[^ ^41^, ^42^ ^]^ which implies that the BHNC structure has the capability to simultaneously detect micro‐nano plastics and other complex pollutants. In addition, the detection limits for SARS‐CoV‐2 S Protein (20–30 nm), small drug molecules MNZ (≈0.5 nm), DA (Dopamine, ≈0.6 nm), AA (Ascorbic Acid, ≈0.7 nm), and MG (Malachite Green, 1–2 nm) are as low as 10^−9^, 10^−7^, 10^−8^, 10^−9^, and 10^−11^ m, respectively, while those for dye molecules R6G (Rhodamine 6G, (1–2 nm) and CV (Crystal Violet, (1–2 nm) are as low as 10^−12^ and 10^−13^ m, respectively, as shown in Figures S15 and S16 (Supporting Information). These molecules exhibit different absorption maxima and Raman shifts. The strong SERS signals confirm the broadband‐enhanced interactions with light in BHNC structures and the superior versatility of the structure. The BHNC structures have excellent linear response, homogeneity, and stability see Figure S16 (Supporting Information). Furthermore, Table S1 (Supporting Information) provides a detailed performance comparison between the proposed structure and typical SERS structures reported in the literature. Due to the PVD process, only the BHNC structure is capable of simultaneous trace detection of micro‐nano plastics, as well as other contaminants.

**Figure 5:**
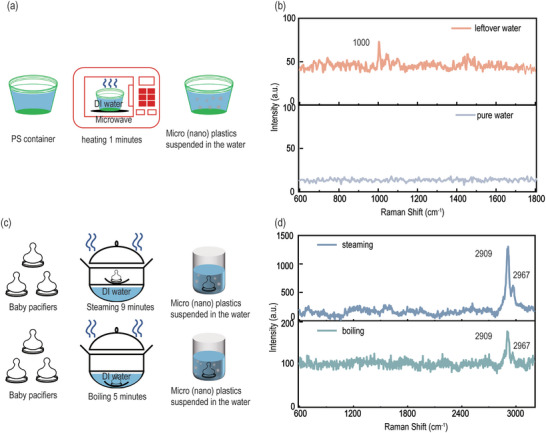
Detection of micro‐nano plastics in real environments by bHNC structure. a) Schematic illustration of the production of micro‐nano plastics in water. b) Raman spectra of the leftover water containing the PS plastics and pure water respectively. c) Schematic diagram illustrating the steaming and boiling process of baby pacifiers in a steamer pot. d) Raman spectra of the baby pacifier micro‐nano plastics in the steaming and boiling water samples respectively.

To further evaluate the validity of the proposed BHNC structure on hierarchical pollutant detection in real‐world scenarios, standard SERS experiments were conducted to monitor micro‐nano plastics released from heated plastic containers and sterilized baby pacifiers, which have been reported in the literature to generate micro‐nano plastics of various sizes.^[^ ^2^, ^43^ ^]^ As shown in **Figure** **5**a, the PS micro‐nano plastics were prepared by filling two‐thirds of PS container with deionized (DI) water to simulate a typical food environment, followed by heating in a microwave oven at 700 W for 1 min.^[^ ^2^, ^44^ ^]^ PS in water is detected by the appearance of a Raman peak at 1000 cm⁻¹, as shown in Figure 5b. The high‐resolution SEM images show that nanometer sized PS particles are well located in the cavity and the micrometer PS is on the surface (Figure S17, Supporting Information). In addition, micro‐nano plastics were also generated near the surface of sterilized baby pacifiers by high‐temperature steam or boiling,^[^ ^43^ ^]^ as shown in Figure 5c. The Raman spectra of the two micro‐nano plastic specimens exhibit the same peaks (Figure 5d), which is consistent with the standard samples reported in the literature.^[^ ^45^ ^]^ The lower Raman intensity of the post‐boiling sample than that of the steam sample indicates the lower plastic concentration in the former, which is induced by the flushing effect of boiling. Using three different brands of baby pacifiers to prepare the plastic solutions by steaming, different Raman signal intensities are observed (Figure S18, Supporting Information), indicating the broad impact or risk of the plastic release on humanity. More detailed SEM images further confirmed the presence of micro‐nano plastics of various sizes on the surface of the pacifier after boiling and steam sterilization, with their locations varying based on size, either inside the cavity or on the surface (Figures S19 and S20, Supporting Information). In addition, the signals of the micro‐nano plastics detected in simulated bottled drinking water after light exposure, nonstick pan use, and incompletely burned disposable food gloves are specifically presented in Figure S21 (Supporting Information). Unfortunately, no micro‐nano plastics were detected in river water, which was highly possible induced by the sediment covering the hotspot, as determined in previous studies. We will address this challenge in our next work. These findings suggest that the BHNC structures can be of promising potential for sensitive photodetection of a wide range of micro‐nano plastics in real‐world environments.

## 2 Conclusion

We proposed a BHNC structure for the detection of micro‐nano plastics and other pollutants in this work. Hierarchical hot spots within the structure (DSHSs, DVHSs, and LHSs) were determined to facilitate the enrichment of target pollutants into optimal hotspots regions, enabling highly sensitive detection of micro‐nano plastics (as low as 10^−8^ g L^−1^), small molecules (down to 10^−13^ m), and SARS‐CoV‐2 S proteins (down to 10^−9^ m) across typical geometry scales ranging from sub‐nanometer to micrometer. Furthermore, our self‐assembled plasmonic structures exhibit broad wavelength SERS response and offer unparalleled sensitivity for the simultaneous detection of a wide range of pollutants in water. Our results provide practical solutions for the unified detection of complex aqueous pollutants in ensuring water environmental safety.

## 3 Experimental Section

### Materials and Chemicals

The anodic aluminum oxide (AAO) templates with V‐shaped nanopores and AAO membranes (13 mm diameter, 5 µm pore size) were purchased from Shenzhen Top Membranes Technology Co., Ltd. Au and Ag particles (5N) were obtained from Zhongnuo Advanced Material Co., Ltd. NaOH was sourced from Aladdin Biochemical Technology Co. Ltd. (Shanghai, China). R6G, MG, CV, DA, AA, and MNZ were purchased from Sinopharm Chemical Reagent Co., Ltd. Silicon dioxide (SiO_2_) was bought from Suzhou Research Material Co., Ltd. Virus was purchased from Sangon Bioengineering (Shanghai) Co. PS micro‐nano plastics with diameters of 30, 50, 100, 300, 500, and 1000 nm supplied as 2.5% (w/v) monodispersed in DI water (18.2 MΩ·cm) were obtained from Rigor Technology Co. Ltd. (Jiangsu, China). Commercial hydrophilic quantitative filter paper (15–20 µm pore size, 13 mm diameter) was supplied by Fuyang Special Paper Co. Ltd. (Zhejiang, China). All reagents were used as received without further purification. DI water (18.2 MΩ·cm) was used throughout the experiments. PS containers and baby pacifiers were purchased from Taobao (https://www.taobao.com).

### Fabrication of the BHNC Structure

The BHNC structure was fabricated by depositing Au onto a V‐AAO substrate using electron beam vapor deposition. The air pressure inside the electron beam chamber was maintained at 8 × 10⁻⁴ Pa, with a coating rate of 3 Å/s, resulting in an Au film thickness of 250 nm. Subsequently, the AAO was removed by etching with a 0.1 M NaOH solution prepared with DI water, and the BHNC was transferred using commercial quantitative filter paper. Finally, Ag was deposited via magnetron sputtering. During the magnetron sputtering process, the air pressure inside the chamber was maintained at 8 × 10⁻⁴ Pa, with a sputtering power of 50 W, and the sputtering duration was 10 min.

### Preparation of PS/ Baby Pacifiers Micro‐Nano Plastic Samples

The PS containers were washed three times with DI water, air‐dried in Petri dishes, filled two‐thirds full with deionized (DI) water, and then placed in a microwave oven and heated to simulate a typical food heating scenario. The container was washed three times with 50 mL of DI water, and large particles in the water were removed through a prefiltration step using an AAO membrane with a 5 µm pore size. Subsequently, 10 mL of the water sample was filtered through the HNC‐Au (250 nm)/Ag. Raman tests were performed after the BHNC‐Au (250 nm)/Ag had dried.

Three popular commercially available brands of silicone baby pacifiers were anonymized using numerical codes (#1‐3), washed three times with DI water, air‐dried in Petri dishes, placed in clean glass Petri dishes, and transferred to a stainless steel steamer basket filled with boiling DI water(heating times according to manufacturer's recommendations); the steamer was covered to maintain the water at 100 °C, and after steaming, the nipples were removed and cooled to room temperature (25 °C), with the washing and collection procedure being the same as above, resulting in an enriched solution volume of 2 mL.

For boiling sterilization, the nipples and glass Petri dishes were fully submerged in boiling DI water for 5 min under the same conditions.

Commercially available mineral water in plastic bottles was irradiated with a xenon lamp for 12 h to simulate sunlight exposure. The xenon lamp provided a full‐spectrum white light source (wavelength: 200–2500 nm) with an intensity of 100 mW cm^−^^2^. After irradiation, large particles formed in the water were first removed using a prefiltration step with an AAO membrane (5 µm pore size). Subsequently, a 10 mL water sample was filtered through the HNC‐Au (250 nm)/Ag, and Raman measurements were conducted after the BHNC‐Au (250 nm)/Ag had dried.

After the non‐stick pan and spatula were cleaned three times with DI water and air‐dried, the spatula was used to stir or scrape the surface of the non‐stick pan continuously for ≈60 s to simulate the stirring process during cooking. Then the non‐stick pan and spatula were washed three times with 50 mL of DI water, and large particles in the water were removed through a prefiltration step using an AAO membrane with a 5 µm pore size. Subsequently, 10 mL of the water sample was filtered through the BHNC‐Au/Ag. Raman tests were performed after the BHNC‐Au/Ag had dried.

The gloves and glass sheet were washed three times with DI water, and air‐dried, and then the gloves were placed on the glass sheet and exposed to the flame generated by a lighter. The gloves were burned for 5–10 s before the flame was extinguished. Then the gloves and glass sheet were washed three times with 50 mL of DI water, and large particles in the water were removed through a prefiltration step using an AAO membrane with a 5 µm pore size. Subsequently, 10 mL of the water sample was filtered through the BHNC‐Au /Ag. Raman tests were performed after the BHNC‐Au /Ag had dried.

### Material Characterizations

SEM and EDS images were obtained using Thermo Fisher Scientific and ZEISS Sigma500 systems.

### Optical Characterizations

Extinction spectra were acquired with a UV–visible (UV–vis) spectrophotometer (UV‐3600). SERS measurements were performed at room temperature using a Raman system (SOL instruments) with an excitation wavelength of 532 nm and a laser power of 13 mW.

### Filtration Performances

The retention efficiency of the BHNC structures for standard micro‐nano plastics was evaluated by the UV‐3600. Subsequently, 2 mL of the standard PS micro‐nano plastics solution was passed through the BHNC structures, and the filtrates were collected. The concentration was calculated using the standard curve, and specific information is provided in the Supporting Information.

### Raman Preparations

Raman spectra for all measurements were collected using single accumulation with a 4 s integration time and a diffraction grating of 750 gr mm^−1^. To ensure data reliability, the average of ten random points in each spectrum was utilized. Small molecules, including R6G, CV, DA, AA, and MNZ, were added dropwise (2 µL) onto the sample surface along with SARS‐CoV‐2 S proteins and allowed to dry before detection. Additionally, 2 mL of micro‐ nano plastic solution was enriched by BHNC structure and dried on the surface for testing.

### Simulations

The Finite‐Difference Time‐Domain method (FDTD) simulations were conducted to calculate the far‐field of the BHNC with different structural designs. The wavelength of the incident light was set at 532 nm along the z‐axis, with the polarization direction oriented along the x‐axis. Periodic boundary conditions were employed. The PS (500 nm) was placed at the center of the HNC‐Au (250 nm)/Ag structure, and the radiated power into the far‐field was simulated.^[^ ^19^, ^46^ ^]^

The finite‐element method (FEM) simulations were conducted to calculate the near‐field of the BHNC with different structural designs. The wavelength of the incident light was set at 532 nm along the z‐axis, with the polarization direction oriented along the x‐axis. Periodic boundary conditions were employed. Structural parameters are provided in the SI.

### Enhancement Factors (EF)

The experimental EF of the HNC‐Au (250 nm)/Ag was expressed as below:^[^ ^7^, ^31^ ^]^

(1) $$EF=\frac{I_{SERS}}{I_{RS}}\;\times \frac{N_{RS}}{N_{SERS}}$$

In this formula, I_SERS_ and I_RS_ represent the peak intensities of the SERS structure and Raman signals on SiO_2_ intensity, respectively. N_SERS_ and N_RS_ were namely the number of probe molecules on the SERS structure and SiO_2_. In this paper, R6G was selected as the probe and the EFs for BHNC‐Au/Ag of the characteristic peak of 612 cm^−1^ were calculated using SiO_2_ as the reference. The SERS signal from 10^−10^ m R6G was analyzed to calculate the enhancement factor (EF).^[^ ^31^ ^]^ For comparison, a 10^−2^ M R6G solution was applied to SiO_2_, and the EF of the BHNC‐Au/Ag was calculated to be ≈ 1.1 × 10⁸, the detailed parameters of the formulae are shown in Note S5 (Supporting Information) and the detailed data are shown in Figure S22 (Supporting Information).

## Conflict of Interest

The authors declare no conflict of interest.

## Supporting information



Supporting Information

## Data Availability

The data that support the findings of this study are available from the corresponding author upon reasonable request.
