# Supplementary material for: Plasmon Enhanced Universal SERS Detection of Hierarchical Plastics by 3D Plasmonic Funnel Metastructure
Source: Adv Sci (Weinh). 2025 May 8;12(23):2500062. doi: 10.1002/advs.202500062 (PMC12199387; doi:10.1002/advs.202500062)
Supplement: Supplementary file 1 — Supporting Information [file ADVS-12-2500062-s001.docx]

**Supplementary information for**

**Plasmon enhanced universal SERS detection of hierarchical plastics by 3D plasmonic funnel metastructure**

Weixi Lu^1^,†, Jian Luo^1,2^,†, Yuyang Zhuang^1^, Jie Liang^1^, Min Xiong^1^, Hui Liu^1^, Lin Zhou^1*^

^1^College of Engineering and Applied Sciences, Nanjing University, Nanjing 210023, China

^2^ School of Physics and Optoelectronic Engineering, Yangtze University, Jingzhou 434023, China

**Contents:**

Note S1. Filtration performance of of the BHNC structure.

Note S2. Details for optical simulation models.

Note S3. Details for the parameter optimization of nanocones.

Note S4. Simulation and experimental analysis of nanocone structures for micro-nano plastics, as well as other pollutants, in various environments.

Note S5. Details for the EF calculations

Supplementary Figures.

Table S1

References

**Note S1 Filtration performance of of the BHNC structure**

Standard curves were generated based on the absorbance at 269 nm of six sizes (30, 50, 100, 300, 500, and 1000 nm) of standard PS micro-nano plastics at various concentrations (Figure S11B). The absorbance of the filtrates was measured at 269 nm, and the concentration was calculated using the standard curve. If the absorbance fell outside the linear range, the solution was appropriately diluted and tested. The retention rate was calculated using Eq.1.

$Rentention rate(\%)=(C_{0}-C_{f})/C_{0} \times100\%$ (1)

where C_0_ and C_f_ represent the concentrations of the original solution and the filtrate, respectively.

**Note S2 Details for optical simulation models**

For the BHNC-Au/Ag, the thickness of the Au film was set at 30.3 nm, while the diameters of the Au NPs and Ag NPs were set at 33.3 nm and 7.9 nm, respectively. Additionally, the spacing between Au NPs and Ag NPs were established at 8 nm and 2.1 nm, consistent with the experimental results.

**Note S3 Details for the parameter optimization of nanocones**

The tilt angle of the structure can effectively regulate the light scattering within the structure as in Figure S6(a), which improves the light-matter interaction and hence the SERS detection capability of the structure. Here, we fixed the radius R1, R2 of the structure and selected three angles to analyze the effect of the tilt angle on the light-substance interaction in the nanocone structure as shown in Figure S6(b). Here, three tilt angles were selected as examples to analyze their effect on the electric field distribution, as shown in Figure S6(c). A larger tilt angle prevents light from fully covering the nanocone, leading to the absence of a hotspot in the corresponding region and consequently reducing the Raman scattering enhancement effect.

Using the preparation method described in the main text, the contrasting structures HNC-Au-13° and HNC-Au-41° were successfully fabricated, as shown in the SEM images in Figure S7(a)-(b). Far-field absorption show that an increase in the tilt angle of the structure enhances light-matter interaction, which is consistent with previous work^1^, as shown in Figure S7(c). The detection results for 10^-7^ M R6G solution and 10^-3^ g/L PS(1μm) solution further confirm that the HNC-Au-21° structure exhibits the best detection performance, as shown in Figure S7(d) and (e).

**Note S4 Simulation and experimental analysis of nanocone structures for micro-nano plastics, as well as other pollutants, in various environments.**

To further theoretically demonstrate the application potential of our BHNC structure, we simulated the electric field distribution and backscattering angle in different practical environments, including air, water, and organic solvents. The results indicate that the hierarchical hotspots of the structure remain effective across these scenarios, with maximum E_max_/E_0_ of 105.1, 53.4, and 53.4, respectively, as shown in Figure S11(a). An electric field enhancement effect is also observed for common micro-nano plastics PET and the contaminant methamidophos (DMF), as shown in Figure S11(b). Additionally, the structure maintains efficient collimation of backscattered light under varying conditions, as shown in Figure S11(c). Finally, the strong potential of the BHNC structure for real-world environmental detection is highlighted by its effective collimation of backscattered light from common pollutants, such as DMF, as well as PET and PS micro-nano plastics, as shown in Figure S11(d).

**Note S5 Details for the EF calculations**

Rhodamine 6G (R6G) were dissolved in water to obtain the solution from 10^-2^ to 10^-13^ M. When the concentrations of R6G are lower than 10^-10^ M, we can only obtain the SERS signal of R6G from the edge of the final evaporation imprint. Thus, to guarantee the scientific nature of the results, the 10^-10^ M R6G solution was chosen as the limit concentration for calculation of the enhancement factor. 2 μL R6G with the concentration 10^-2^ and 10^-10^ M were dropped separately on the SiO_2_ substrate and fabricated SERS substrates. Before carrying out the Raman detection, the samples were all totally dried. The estimated maximum diameter of the final evaporation imprint on SiO_2_ substrate were around 2.5 mm for R6G (10^-2^ M). And the estimated maximum diameter of the final evaporation imprint was around 2 mm for R6G (10^-10^ M) for the hydrophobic property of the fabricated substrate. Thus, the average areal density (AD) of the R6G can be estimated by the following equation: where and represent the volume of the analytes solution dropped, the concentration of analytes solution, Avogadro constant and the area of the analytes molecules covered, respectively.

With the R6G concentration of 10^-10^ M, the AD is around 38.3 molecules/μm^2^.

The enhancement factor was estimated by the following equation: $\text{EF}=(I_{\text{SERS}}\times N_{SiO_{2}})/(I_{SiO_{2}}\times N_{SERS})$ , where $I_{SERS}$, $I_{SiO_{2}}$, $N_{SiO_{2}}$ and $N_{SERS}$ represent the intensity of SERS signal, Raman signal intensity obtained from SiO_2_, the number of analytes molecules within laser spot on SiO_2_ substrate and the number of molecules within laser spot on SERS substrate, respectively. The Raman spectrometer was all used under the condition (13 mW laser power, ×10 objective lens, 1 μm laser spot). The Raman signal intensity of R6G (10^-10^ M) obtained from bi-metallic hollow nanocones (BHNC) SERS substrate, R6G (10^-2^ M) and collected from SiO_2_ substrate are shown in Figure 22. Thus, the average EF of the BHNC substrate for R6G can be obtained.

Thus, the average areal density (AD) of the R6G can be estimated by the following equation: where and represent the volume of the R6G solution dropped, the concentration of R6G solution, Avogadro constant and the area of the R6G molecules covered, respectively.

With the R6G concentration of 10^-10^ M, the AD is around 38.3 molecules/μm^2^.

With the R6G concentration of 10^-2^ M, the AD on SiO_2_ is around 2.45*10^9^ molecules/μm^2^.


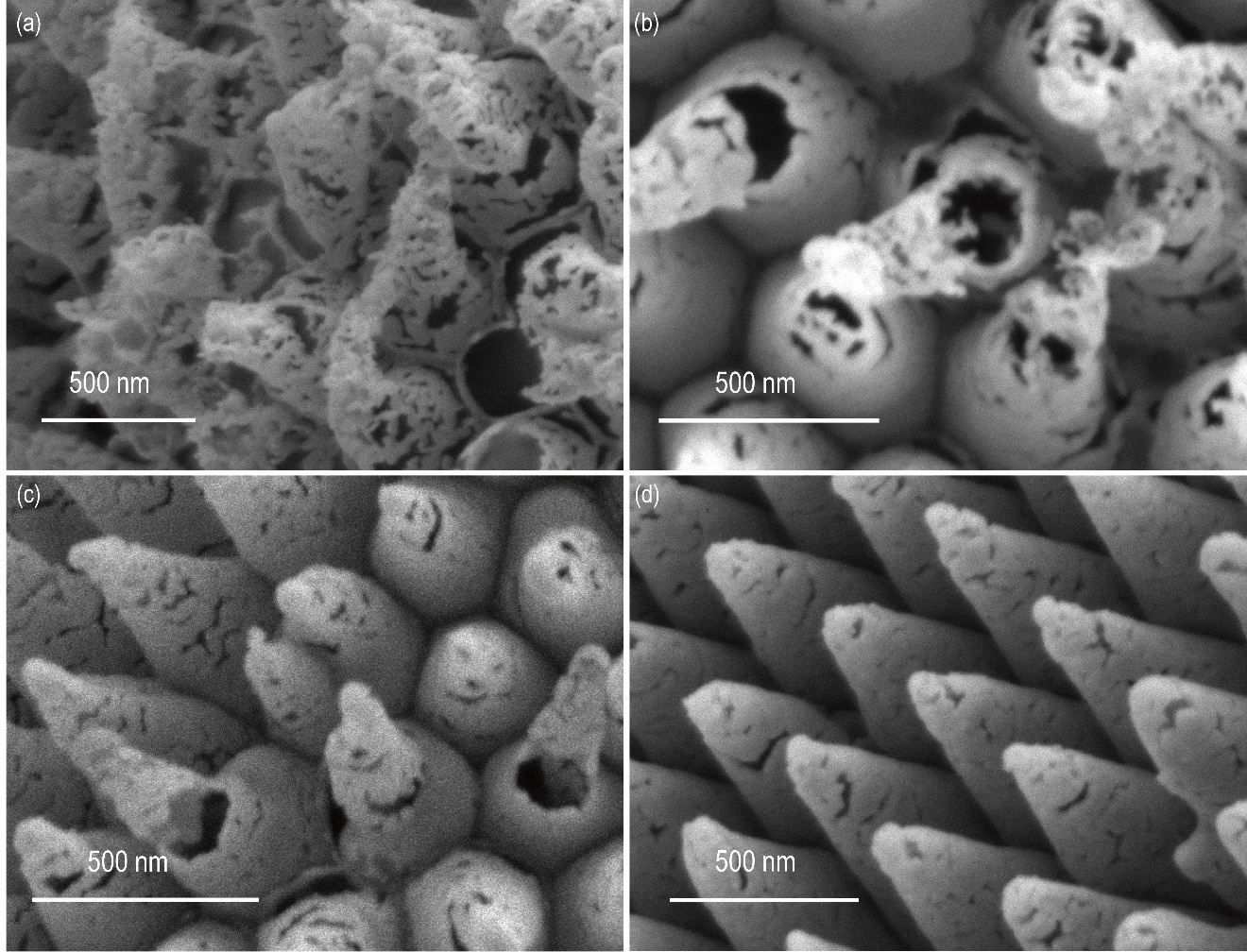


**Figure S1. (a-d) 3D cross-section of BHNC-Au (100, 150, 200, 300 nm)/Ag (30 nm) where the deposition rate of Au is 5 A/S.**


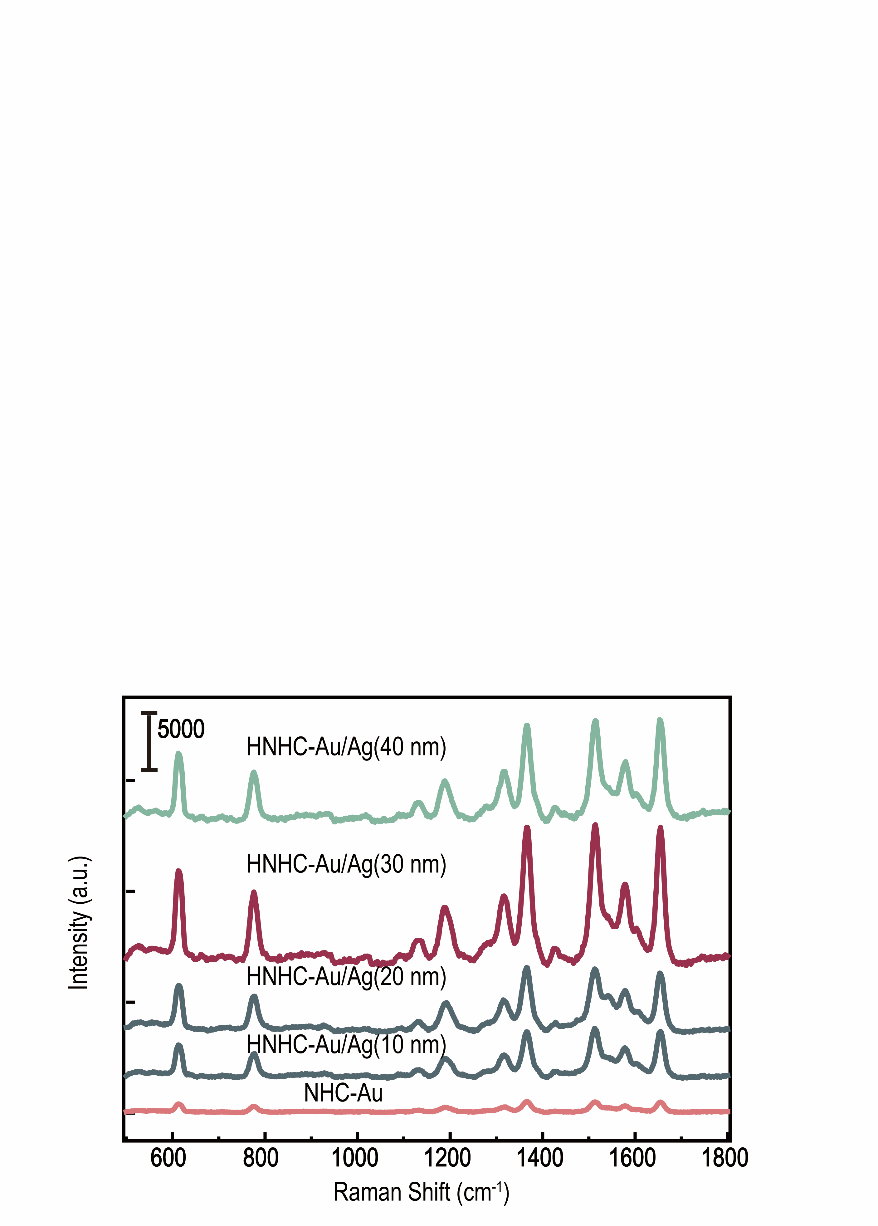
**Figure S2. Raman spectra of R6G (10^−7^ M) collected from BHNC-Au with different Ag thicknesses.**


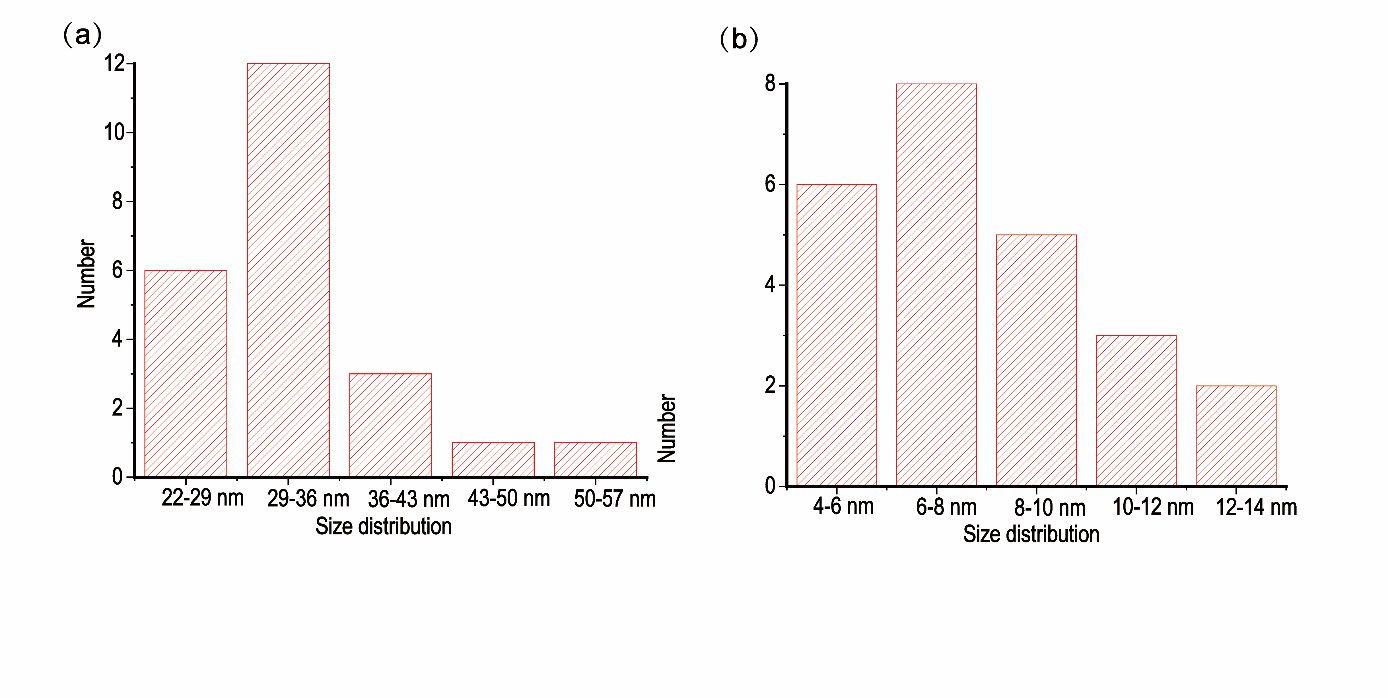


**Figure S3. Statistical distribution of Au NPs and Ag NPs sizes in BHNC-Au/Ag structures.**


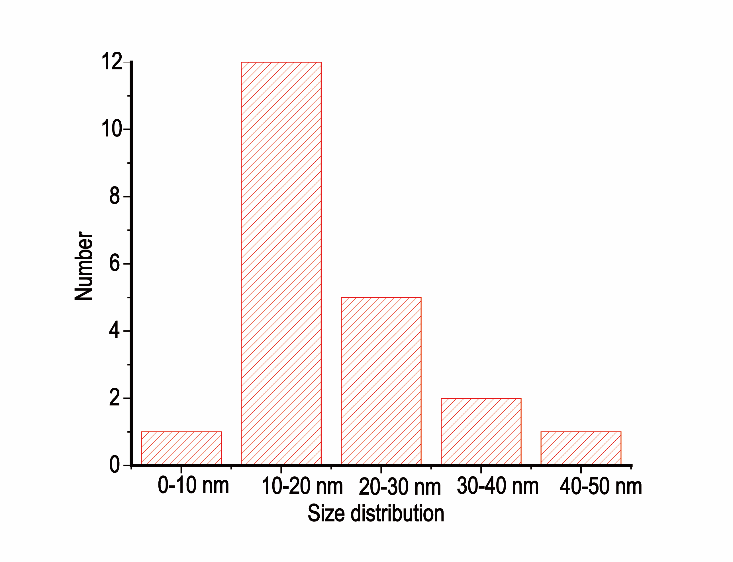


**Figure S4. Statistical distribution of intergap size in BHNC-Au/Ag structures.**


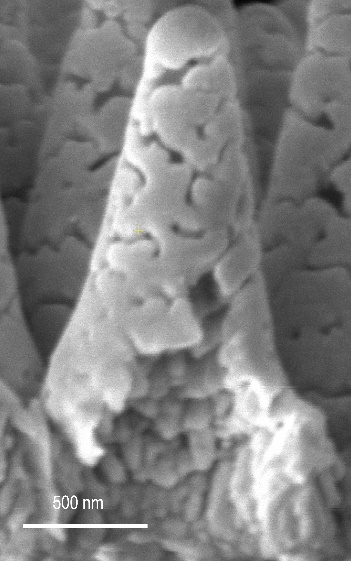


**Figure S5. SEM image of the cross-section of BHNC-Au/Ag structures.**


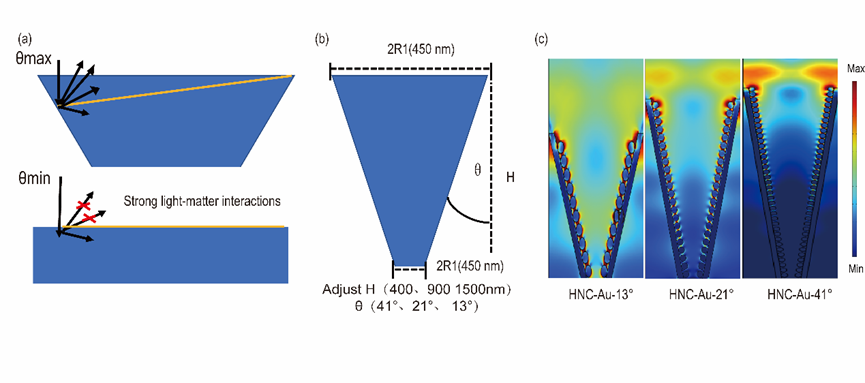


**Figure S6. (a) Schematic diagram of structural parameters. (b) Theoretical limit orientation diagrams. (c) Near-field electric field distribution of HNC at a wavelength of 532 nm.**


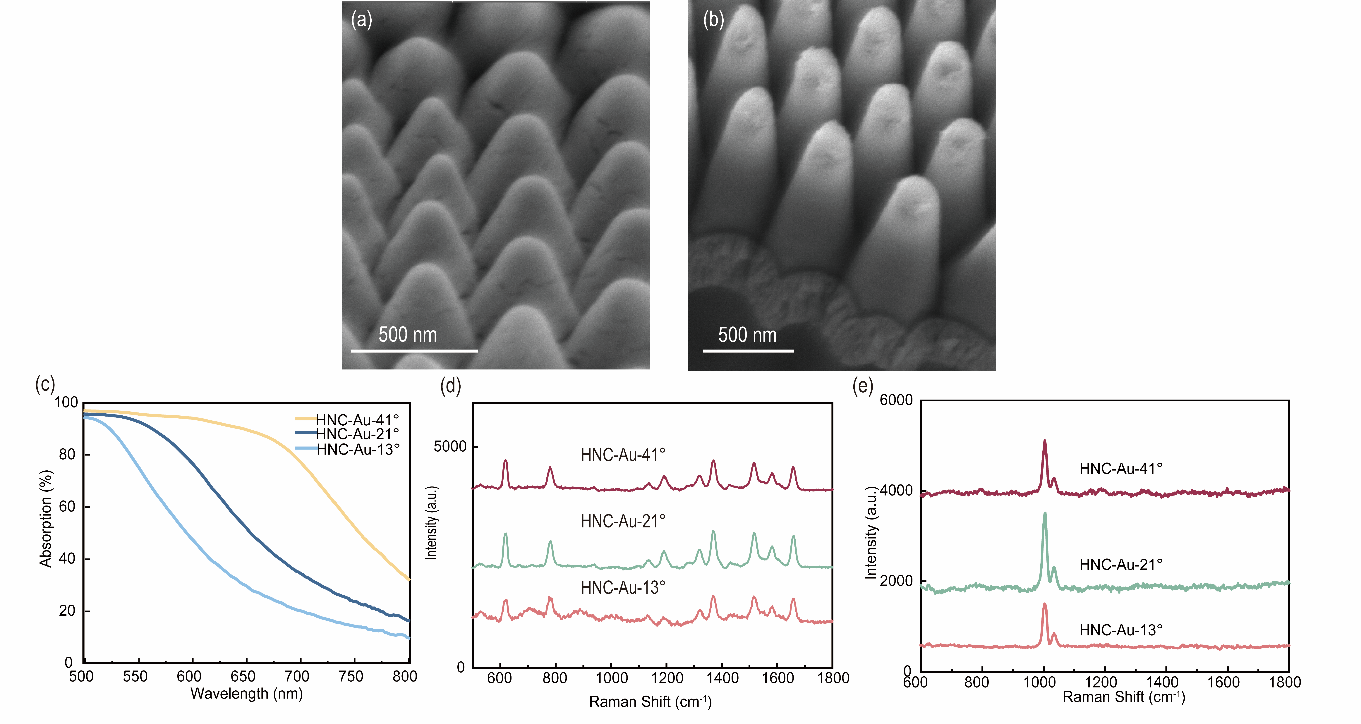


**Figure S7. (a, b) 3D cross-sectional SEM images of the prepared HNC structures with tilt angles of 13° and 41°, respectively. (c) Far-field absorption spectra of HNC at different tilt angles. (d) Raman spectrum of R6G at 10^-7^ M and (e) Raman spectrum of PS (50 nm) at 10^-3^ g/L on HNC structures.**


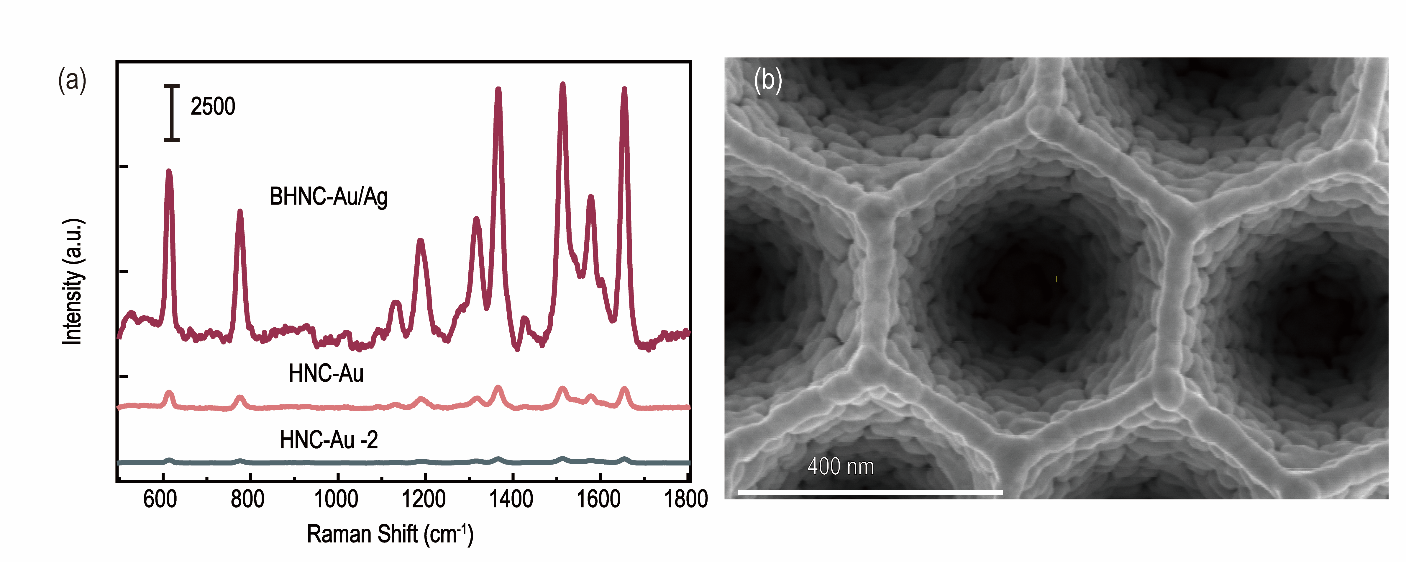


**Figure S8.** **(a) Raman spectra of R6G with concentrationof 10^−7^ M obtained from the three type of HNC structures. (b) SEM image of the HNC-Au-2 structure, showing that the Au NP size and gaps are similar to those in the HNC-Au structure.**

s


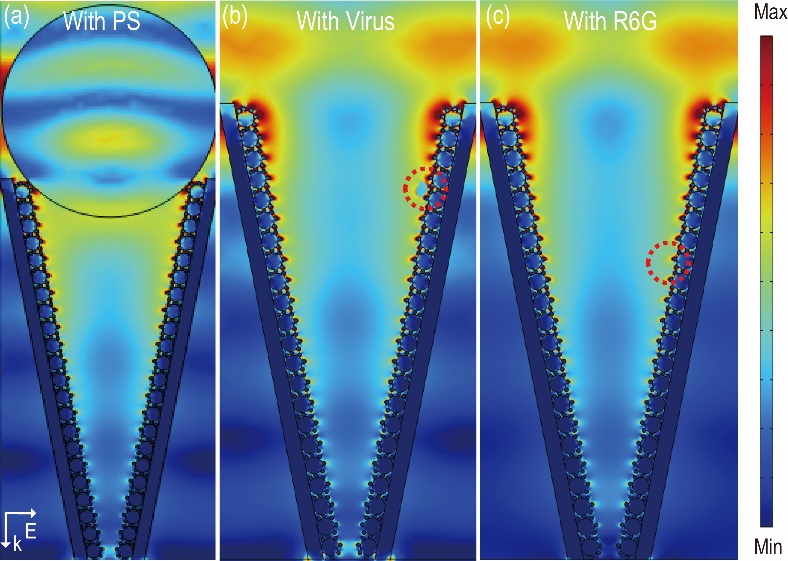


**Figure S9.** **Electric field distribution views for BNHC-Au/Ag with (a) PS, (b) Virus, and (c) R6G at a wavelength of 532 nm, respectively.**


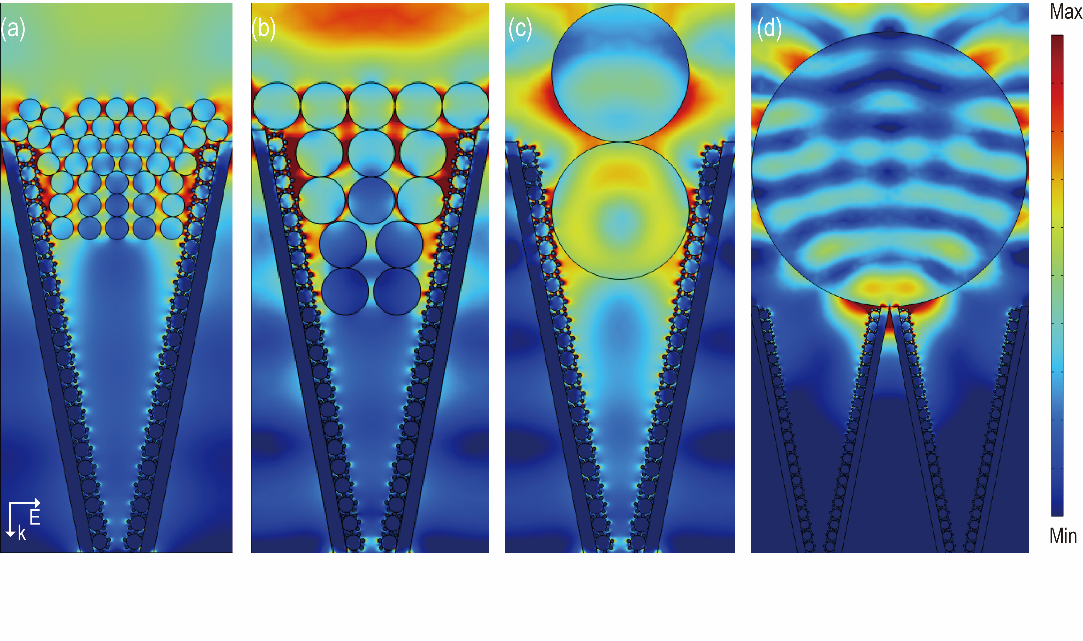


**Figure S10.** **Electric field distribution views for BNHC-Au/Ag with (a) 50 nm PS, (b) 100 nm, (c) 300 nm, (d) 1000 nm at a wavelength of 532 nm, respectively.**

**
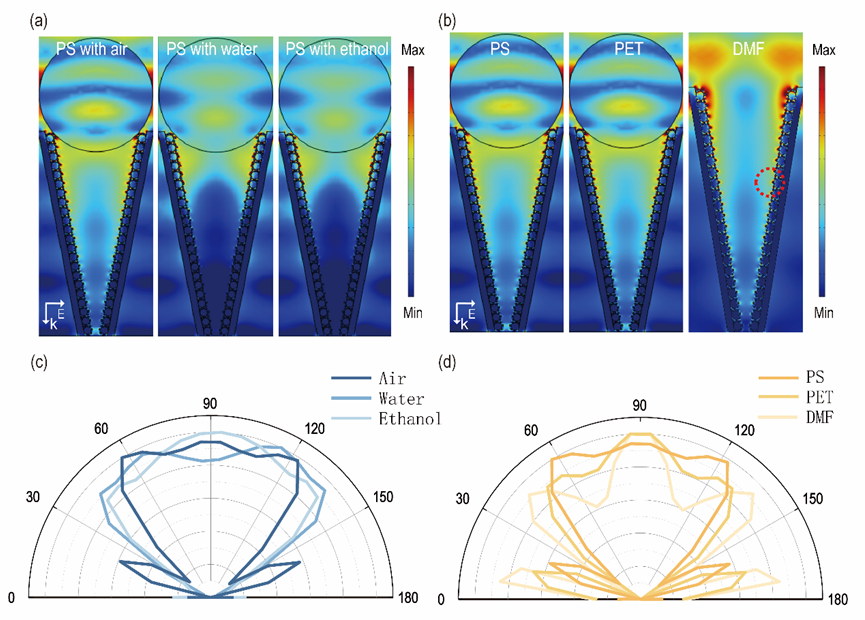
**

**Figure S11.** **(a) Near-field electric field distribution of BHNC in air, water, and ethanol at a wavelength of 532 nm. (b) Near-field electric field distribution of BHNC with PS, PET, and DMF at a wavelength of 532 nm. (c) Normalized far-field radiation patterns of BHNC in air, water, and ethanol. (d) Normalized far-field radiation patterns of BHNC with PS, PET, and DMF.**


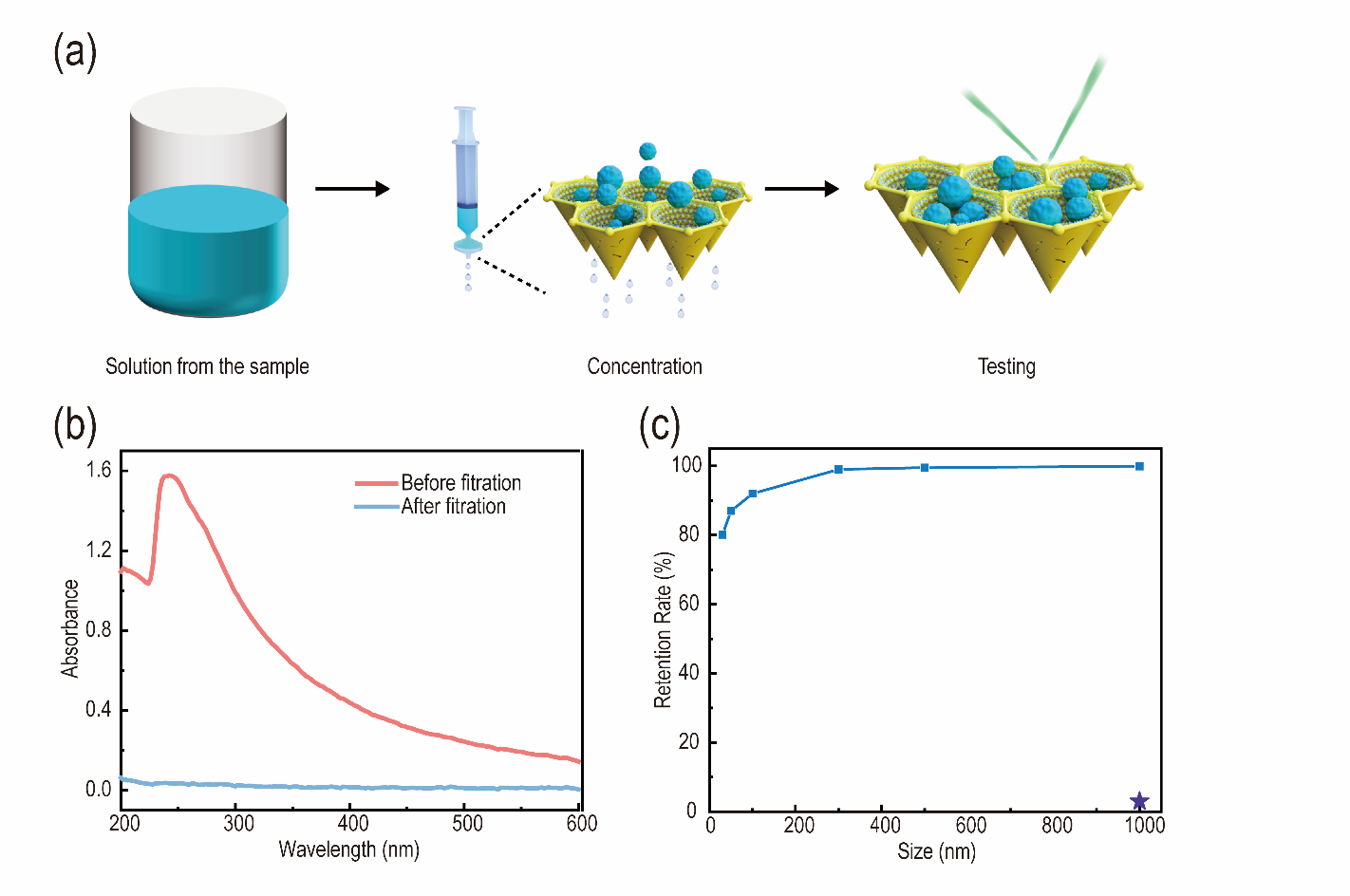


**Figure S12. (a)** **The schematic of the enrichment effect. (b) UV–vis absorption spectra of 300 nm standard PS nanoplastic solution (0.1 g/L)before and after filtration. (c) Retention rate of standard PS micro-nano plastics of various sizes, and retention of 1000 nm PS plastics on commercial filter paper alone (represented by pentagrams).**


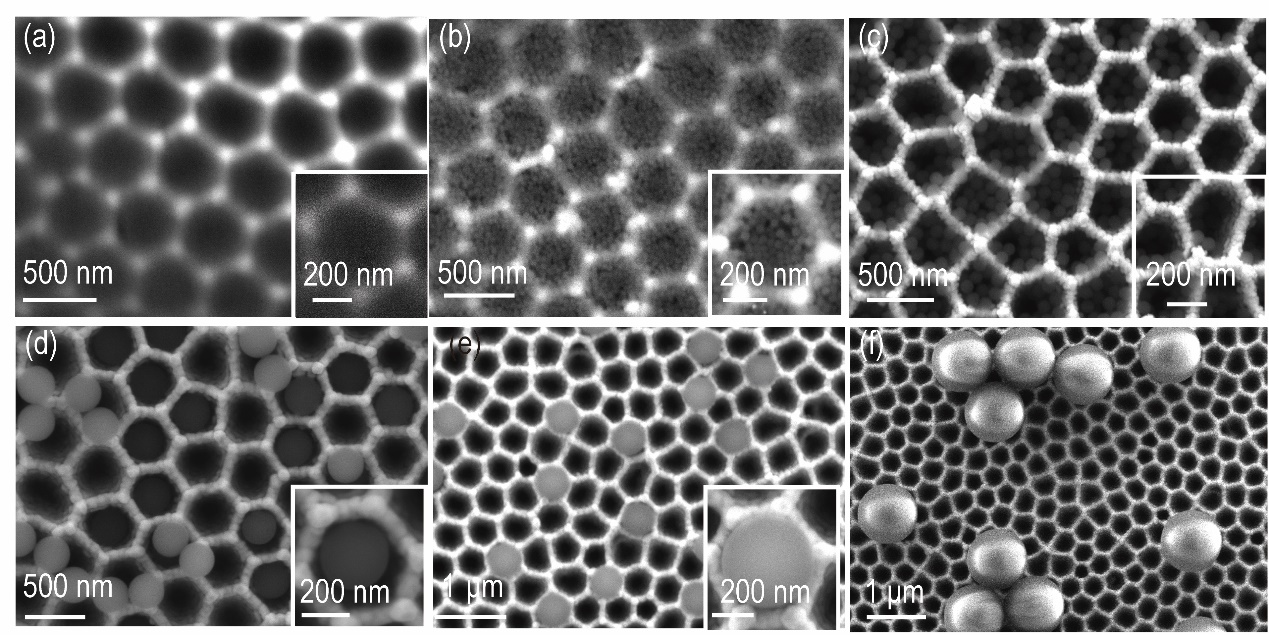


**Figure S13. SEM images of BHNC-Au/Ag structures capturing micro-nano plastics of various sizes.**


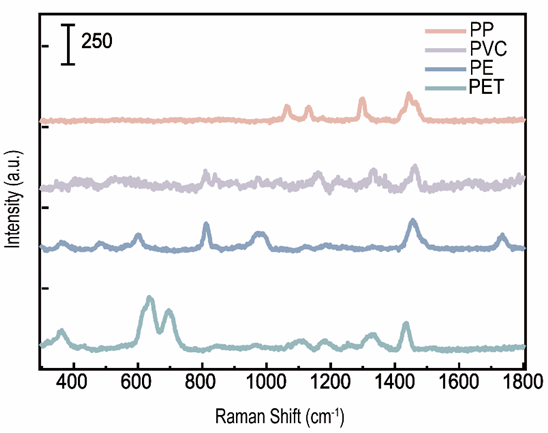


**Figure S14. The BHNC structures SERS spectra of PP, PVC, PE, and PET at 10^-3^ g/L(1 μm).**


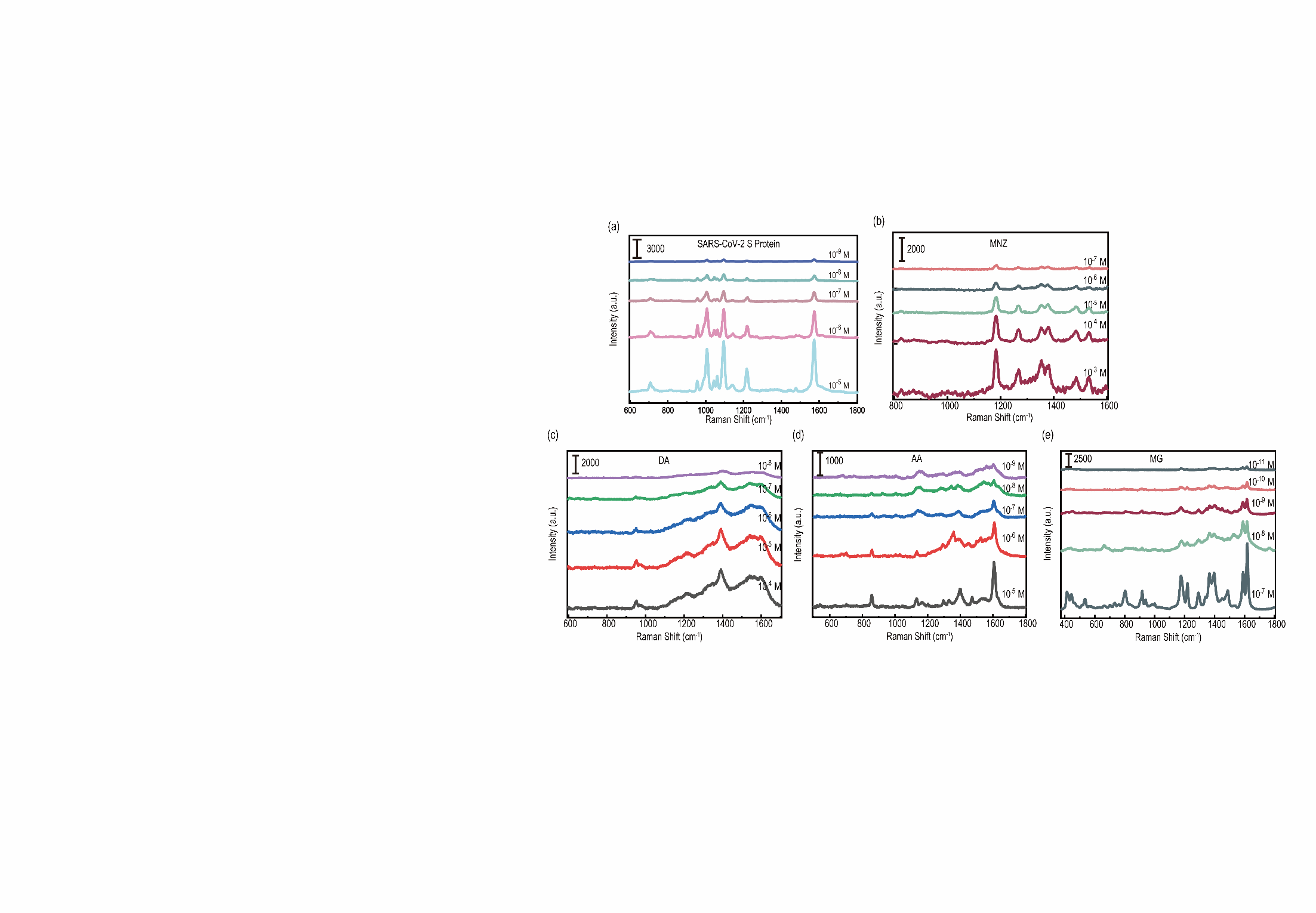


**Figure S15.** **(a)–(d) BHNC-Au/Ag structures SERS spectra of DA, MNZ, MG, and AA at varying concentrations.**


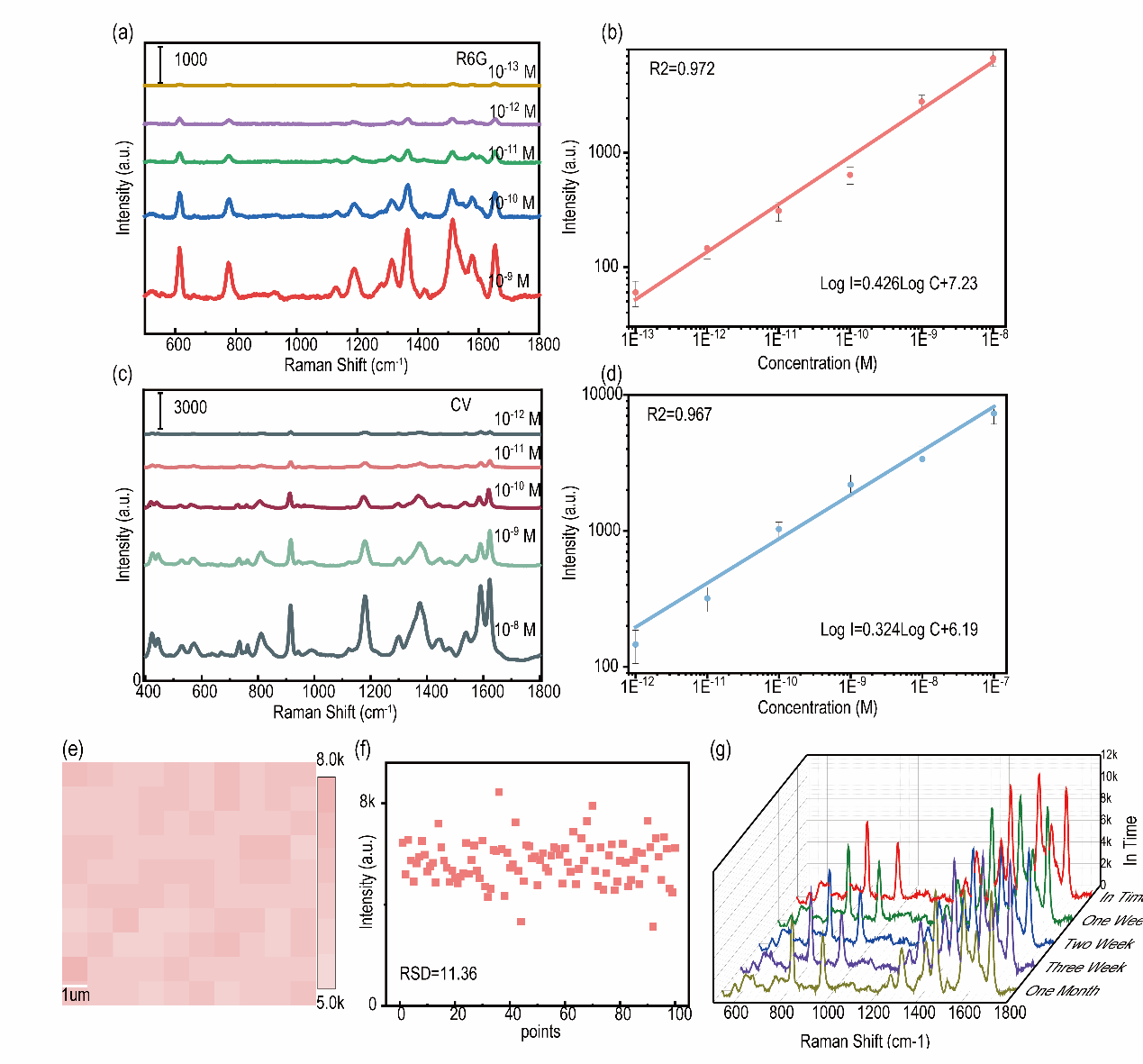


**Figure S16.** **(a) SERS spectra of R6G at varying concentrations. (b)** **Linear fitting lines of R6G(612 cm^−1^).** **(c) SERS spectra of CV at varying concentrations. (d)** **Linear fitting lines of CV(1619cm^−1^). (e) Raman mapping of R6G (10^−7^ M) collected from BHNC-Au /Ag. (f) The RSD values of 612 cm^−1^ bands collected from BHNC-Au/Ag. (g) Raman spectra of R6G derived from BHNC-Au/Ag substrate for one month.**


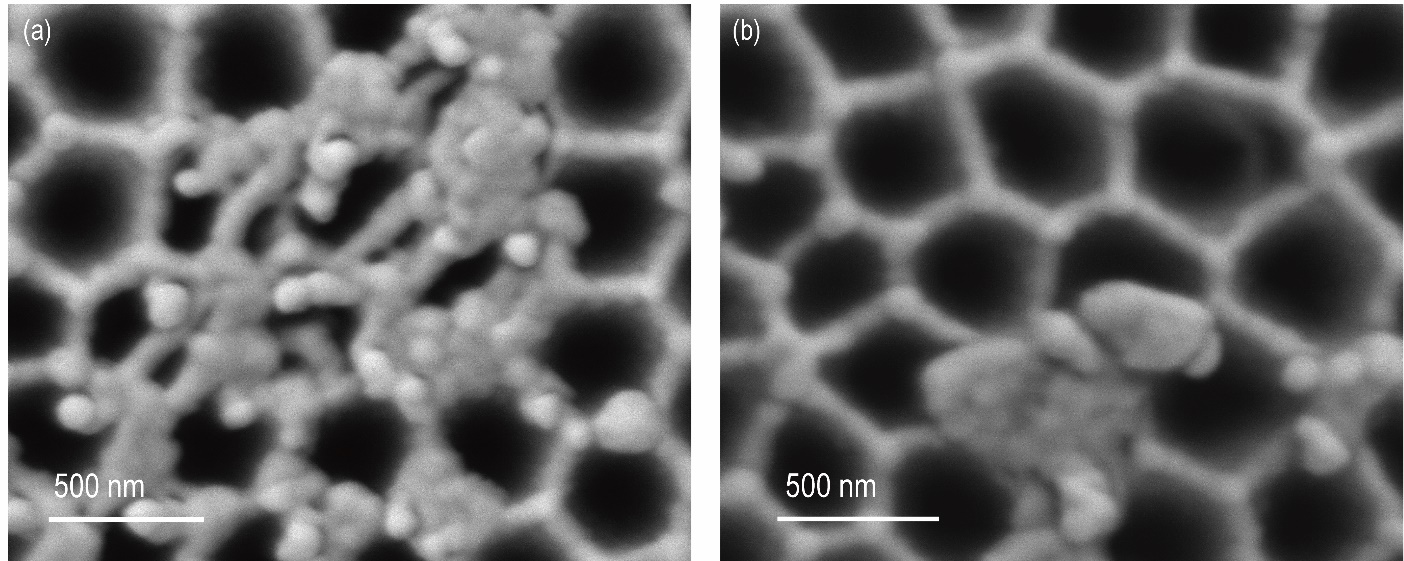


**Figure S17. SEM images of PS (a) microplastics and (b) nanoplastics retained on the BHNC-Au/Ag.**


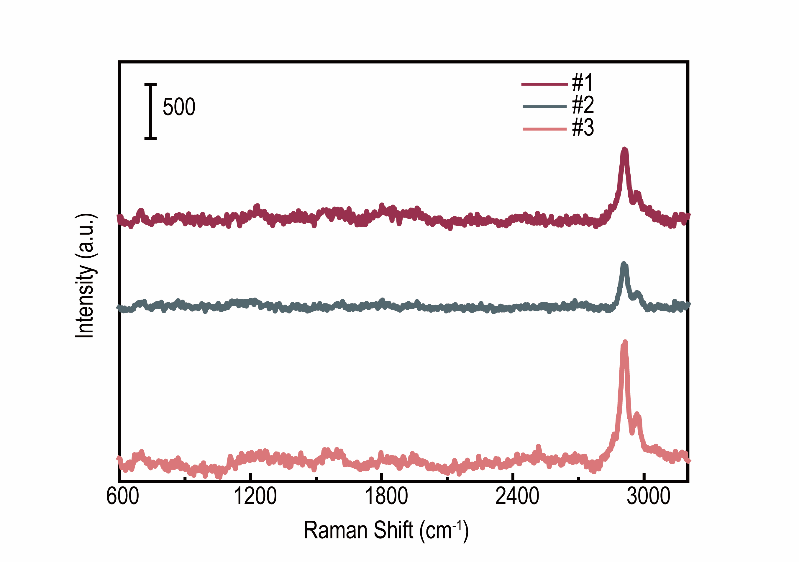


**Figure S18.** **Raman spectra of micro-nano plastics from the three brands of baby pacifiers in the steamed samples.**


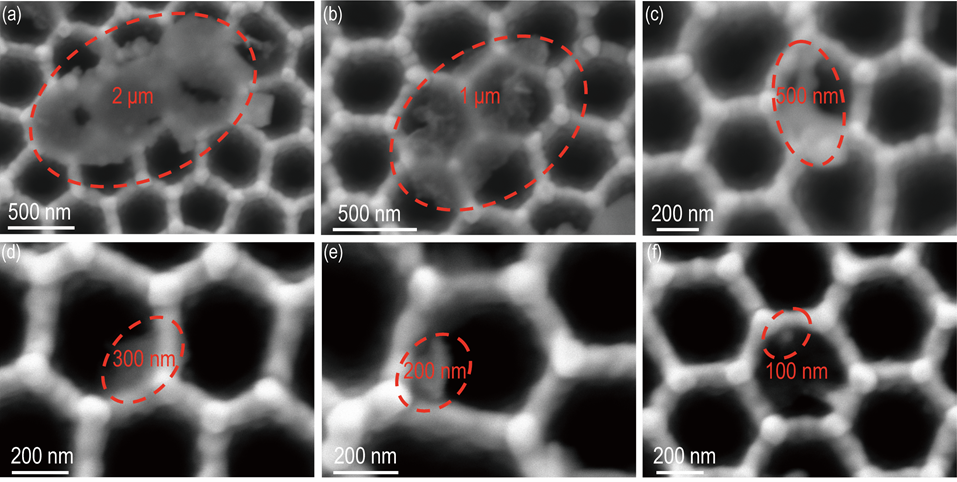
**Figure S19. SEM images of baby pacifier microplastics (a, b) and nanoplastics (c-f) retained on BHNC-Au/Ag samples after boiling sterilization.**


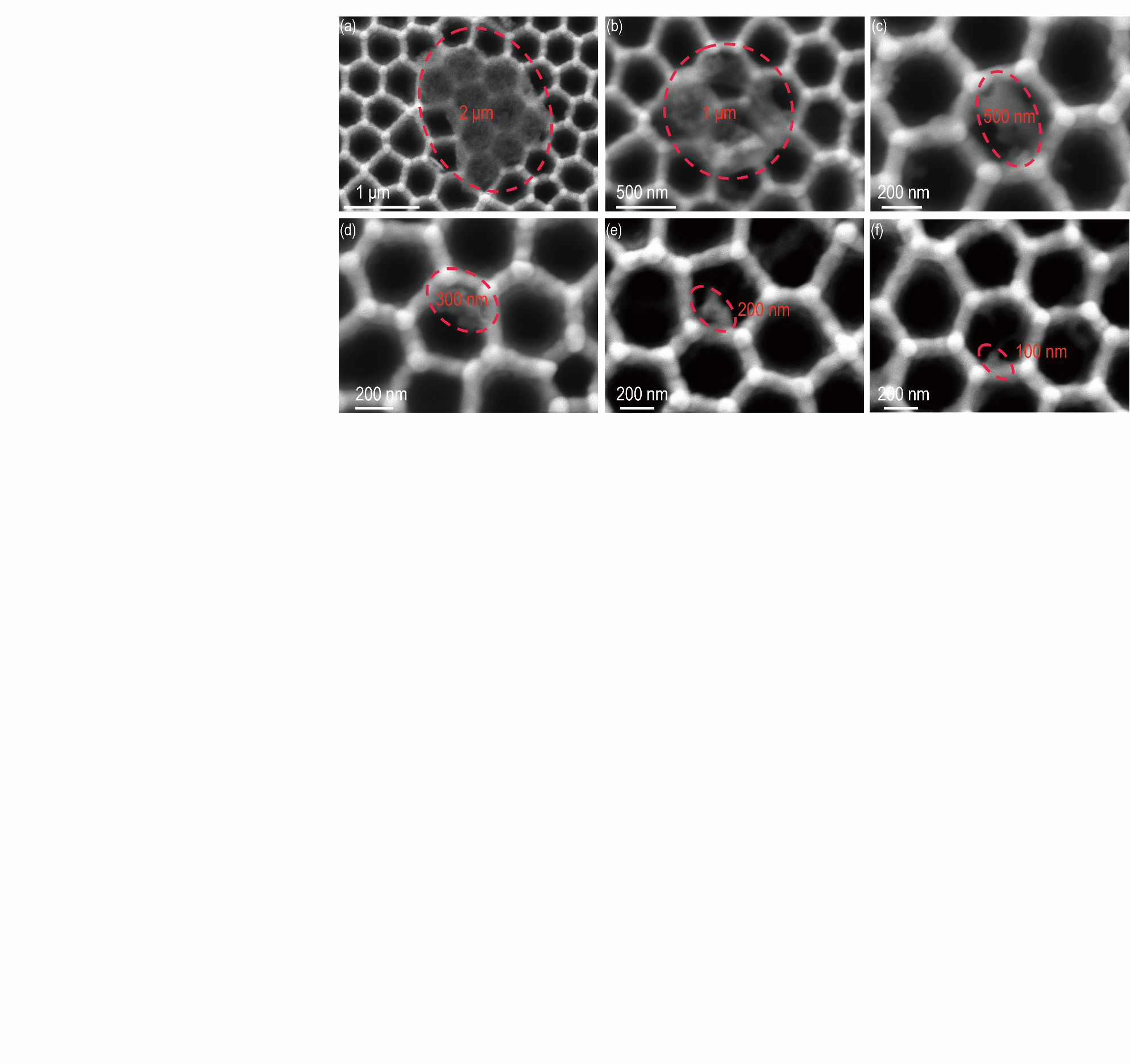


**Figure S20. SEM images of baby pacifier microplastics (a, b) and nanoplastics (c-f) retained on BHNC-Au/Ag samples after** **steam sterilization.**


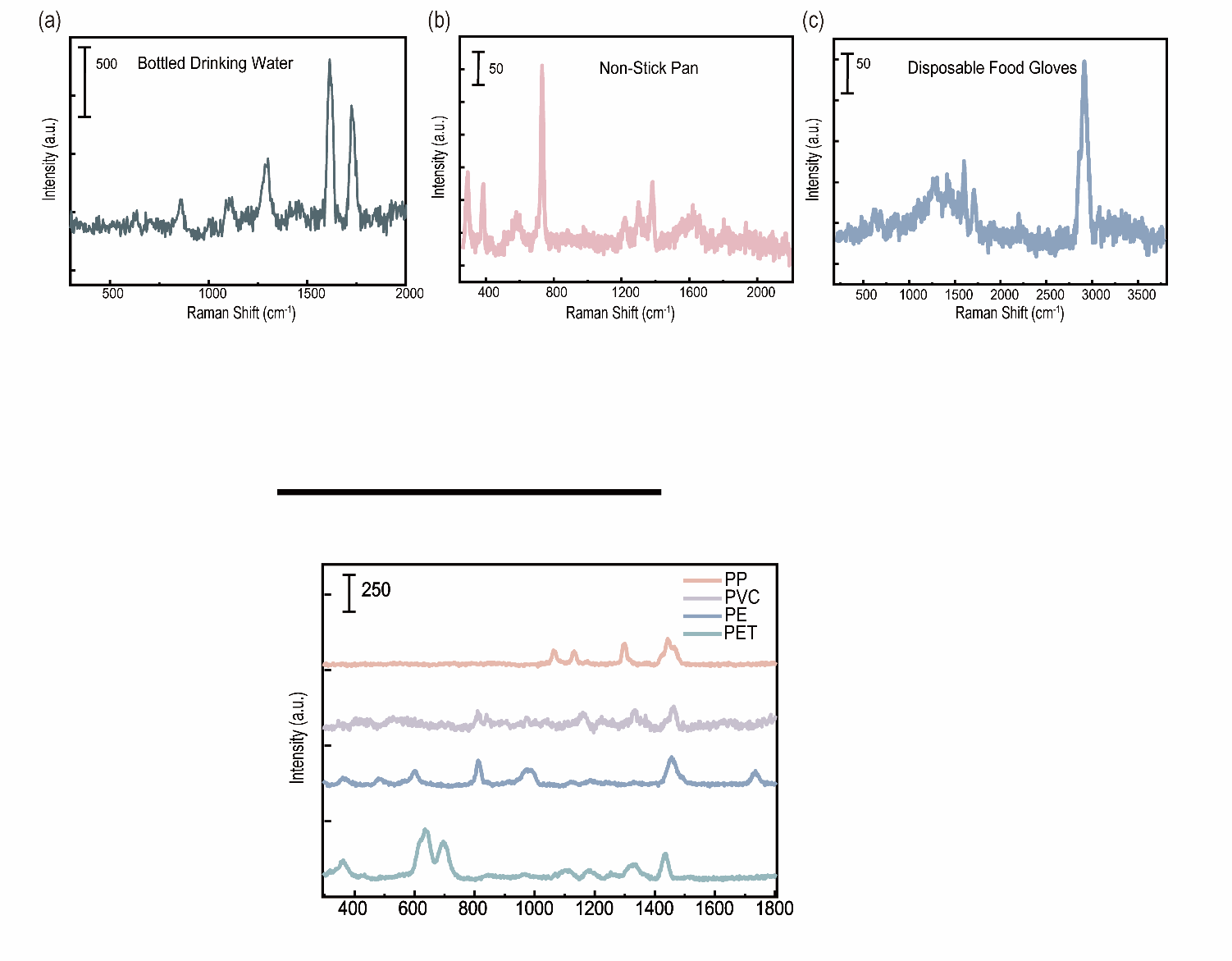


**Figure S21. Raman spectra of solutions of (a) bottled drinking water after simulated light exposure, (b) a non-stick pan rinsed with deionized water after stirring and (c) incompletely burned disposable food safety gloves rinsed with pure water.**


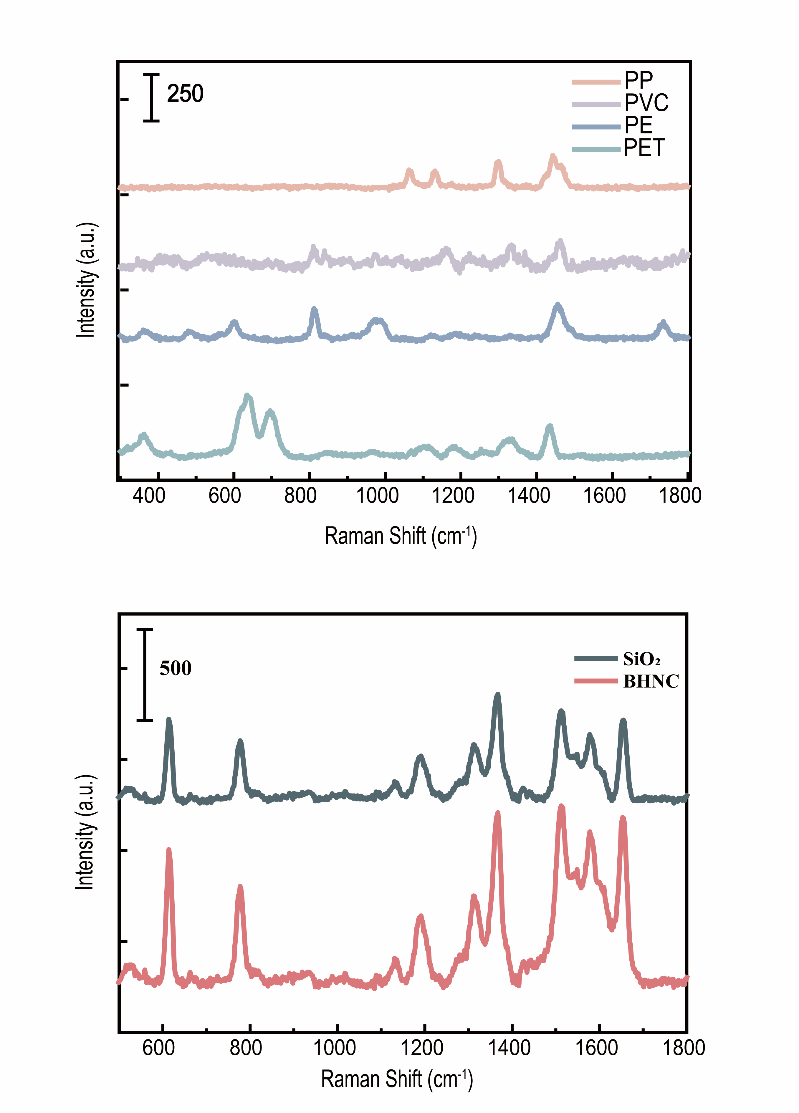


**Figure S22. The SERS spectra of R6G at 10⁻¹⁰ M on BHNC-Au/Ag structures and R6G at 10⁻² M on SiO₂.**

**Table S1.** Sensitivity performance comparison.

| **Structure** | **Molecular** | **CONC** | **Virus** | **CONC** | **Nanoplastics** | **CONC** |
| --- | --- | --- | --- | --- | --- | --- |
| This work | R6G  (< 2nm) | 10^-13^ M | SARS-CoV-2 S Protein (20-30 mn)  (20-30 nm) | 10^-9^ M | PS  (30-1000 nm) | 10^-8^ g/L |
| Ag/graphene/ZnO^2^ | R6G | 10^-9^ M | SARS-CoV-2 S Protein | 4×10^-9^ M | PS  (50 nm) | 5×10^-2^ g/L |
| PIQC ZnO/Ag^3^ | R6G | 10^-18^ M | \ | \ | \ | \ |
| AAO/MoS_2_/Ag cavities^4^ | R6G | 10^-11^ M | \ | \ | PS  (100-300 nm) | 10^-3^ g/L |
| Ta_2_C MXene^5^ | MeB  (< 2nm) | 10^-7^ M | SARS-CoV-2 S Protein | 10^-9^ M | \ | \ |
| AgNPs/SiNWs^6^ | R6G | 2×10 ^-6^ M | SARS-CoV-2 S Protein | 9.3×10^-12^ M | \ | \ |
| Ag nanowire membrane^7^ | PATP  (< 2nm) | 10^-7^ M | \ | \ | PS  (50-1000 nm) | 10^-7^ g/L |
| GO/MWCNT–AgNS membrane^8^ | 4-Pyridinethiol  (< 2nm) | 10^-8^ M | \ | \ | PS  (50-1000 nm) | 10^-5^ g/L |

\ represents the tests that were not performed for the work.

**Table S1** illustrates the performance comparison between the proposed structure and other SERS structures. While the PS detection concentrations reported in references^7,8^ reached 10^-5^ g/L and 10^-7^ g/L, respectively, the addition of substances like KI makes it impossible to detect small molecules.As demonstrated, the structure in this work not only enables the simultaneous detection of multiple substrate from nanometer to micrometer scale, but also maintains a relatively high sensitivity.

**REFERENCES**

1 Mao, P. *et al.* Broadband single molecule SERS detection designed by warped optical spaces. *Nature communications* **9**, 5428 (2018).

2 Du, B. Q. *et al.* Thermoelectrically Driven Dual-Mechanism Regulation on SERS and Application Potential for Rapid Detection of SARS-CoV-2 Viruses and Microplastics. *Acs Sensors* **9**, 502-513 (2024).

3 Yu, J. *et al.* Hierarchical Particle-In-Quasicavity Architecture for Ultratrace Raman Sensing and Its Application in Real-Time Monitoring of Toxic Pollutants. *Anal Chem* **92**, 14754-14761 (2020).

4 Li, J. *et al.* Particle-in-molybdenum disulfide-coated cavity structure with a Raman internal standard for sensitive Raman detection of water contaminants from ions to< 300 nm nanoplastics. *The Journal of Physical Chemistry Letters* **13**, 5815-5823 (2022).

5 Peng, Y. S. *et al.* Charge-Transfer Resonance and Electromagnetic Enhancement Synergistically Enabling MXenes with Excellent SERS Sensitivity for SARS-CoV-2 S Protein Detection. *Nano-Micro Lett* **13**, 52 (2021).

6 Daoudi, K. *et al.* Ultra-sensitive and fast optical detection of the spike protein of the SARS-CoV-2 using AgNPs/SiNWs nanohybrid based sensors. *Surf Interfaces* **27**, 101454 (2021).

7 Yang, Q. *et al.* Identification of Trace Polystyrene Nanoplastics Down to 50 nm by the Hyphenated Method of Filtration and Surface-Enhanced Raman Spectroscopy Based on Silver Nanowire Membranes. *Environ Sci Technol* **56**, 10818-10828 (2022).

8 Jiang, Y. *et al.* Silver nanostars arrayed on GO/MWCNT composite membranes for enrichment and SERS detection of polystyrene nanoplastics in water. *Water Res* **255**, 121444 (2024).
